# Single-cell transcriptome profiling reveals herpesviral manipulation of host processes in spleen of gibel carp infected with a *Cyvirus cyprinidallo2*

**DOI:** 10.1371/journal.ppat.1014114

**Published:** 2026-04-02

**Authors:** Fei Ke, Yu Zhou, Bo-Qian Chen, Han-Yue Wang, Zhe Zhao, Qi-Ya Zhang

**Affiliations:** 1 State Key Laboratory of Breeding Biotechnology and Sustainable Aquaculture, Institute of Hydrobiology, Chinese Academy of Sciences, Wuhan, China; 2 Jiangsu Province Engineering Research Center for Marine Bio-resources Sustainable Utilization, College of Oceanography, Hohai University, Nanjing, China; University of Southern California, UNITED STATES OF AMERICA

## Abstract

The herpesviral haematopoietic necrosis with acute gill hemorrhage has recently become prevalent in gibel carp farming regions and has resulted in significant losses. Although several herpesvirus strains (belonging to *Cyvirus cyprinidallo2*) have been isolated, the permissive cells *in vivo*, how the virus manipulates host cell processes, and causes disease remain largely unclear. In this study, the isolate *Carassius auratus* herpesvirus (CaHV) was used as a model to dissect the interactions between virus and host through single-cell RNA sequencing (scRNA-seq) and functional validation. Spleen of the infected fish was selected for scRNA-seq analysis for its highest viral load, which resolved splenic cells into ten distinct clusters, and macrophages and mural cells were identified as the major viral permissive cells. By classification of the permissive cells into infected and bystander cells, we found that CaHV induced widespread suppression of host cellular processes in infected cells, especially the antiviral responses. Meanwhile, negative immune regulators and factors potentially involved in viral entry, assembly, and egress, including heparan sulfate and the ESCRT complex, were upregulated to facilitate virus infection. Further sub-clustering and pseudotime inference analysis revealed distinct macrophage subpopulations, while novel subsets emerged with CaHV infection. Macrophage subsets toward M2-like polarization were also observed. In addition, enrichment and apoptosis analyses revealed that the hemorrhagic symptoms in diseased fish resulted from a combination of virus-induced coagulation dysfunction and endothelial cell programmed cell death. Collectively, the present study provides comprehensive new insights into the targeted cells of *Cyvirus cyprinidallo2* and its interactions with the host, while also identifying potential targets for disease control.

## Author summary

Herpesviruses of the genus *Cyvirus* (family *Alloherpesviridae*) have caused significant losses in aquaculture. The *Cyvirus cyprinidallo2* isolate has become the most serious pathogen of gibel carp. The specific cell types targeted by *Cyvirus cyprinidallo2* and the ways the virus manipulates cellular processes for replication and pathological changes have remained largely unknown. In this study, scRNA-seq analysis of spleen samples from CaHV (an isolate of *Cyvirus cyprinidallo2*) infection revealed that the main permissive cells *in vivo* are macrophages and mural cells. Further research confirmed that CaHV suppressed several host responses, including IFN responses, and upregulated genes involved in immune negative regulation and in viral entry, assembly, and egress, which could represent one of the strategies for the viral replication in infected cells. We also observed that CaHV infection induced the emergence of new macrophage subpopulations and drove macrophages toward an anti-inflammatory (M2) state. Additionally, we found that coagulation and anticoagulation dysfunction, along with programmed cell death of vascular endothelial cells, are the main reasons for virus-induced hemorrhaging. These data provided a comprehensive understanding of the cellular landscape in the spleen of a *Cyvirus cyprinidallo2*-infected host, reveal several antiviral targets, and offer references for other fish herpesviruses.

## Introduction

Herpesviruses constitute a large viral order (Herpesvirales) with a broad host range covering higher vertebrates, lower vertebrates, and invertebrates [1,2]. Among them, the most well-known are mammalian-infecting herpesviruses, such as herpes simplex virus (HSV) and Varicella-Zoster virus (VZV) [3]. The order Herpesvirales comprises three families, with fish- and frog-infecting herpesviruses classified under *Alloherpesviridae* [1,2]. Cyprinid fish—including common carp, crucian carp, gibel carp, goldfish, and koi—are economically significant for aquaculture and ornamental purposes, with combined annual production of common carp, crucian carp, and gibel carp nearing 7 million tons [4]. However, these species face severe threats from herpesviruses of the family *Alloherpesviridae*, notably the isolates of *Cyvirus cyprinidallo1*, *2*, and *3* [5,6] in the genus *Cyvirus*, some of which have been listed by the World Organization for Animal Health (WOAH) in its Animal Diseases directory.

*Cyvirus cyprinidallo2* has been recognized worldwide as an important pathogen in species of the genus *Carassius* [2,7]. It causes a disease originally named herpesviral hematopoietic necrosis (HVHN) [8]. Over the past decade, it has become the main causative agent of gibel carp (*C. gibelio*) in the aquaculture industry, an amphitriploid subspecies widely cultivated in China [9,10]. Currently isolated viral strains possess a genome of approximately 275–290 kbp, and encode around 150 open reading frames (ORFs) [11–13]. A typical symptom in diseased gibel carp is gill hemorrhage, seen as massive bleeding when the operculum opens [14]. Another clinical sign includes hemorrhagic spots of varying sizes on the skin, especially on the lower jaw, fin bases, and areas below the lateral line scales [15]. Several research advances have been made, including genome and coding potential characterization [7,11,12,16,17] and studies on virus entry and induced host responses [18–21]. However, critical knowledge gaps, including the virus-targeted cells *in vivo*, remain unknown. Further research is also urgently needed to elucidate how the virus manipulates host defenses to facilitate its propagation and drive pathological changes. These insights are key to developing effective control strategies.

Single-cell RNA sequencing (scRNA-seq) technology is a cutting-edge method that enables high-throughput transcriptomic profiling at the single-cell level. By analyzing individual cells, this technique uncovers cell-to-cell heterogeneity, revealing unique gene structures and expression patterns that are masked in bulk tissue analyses [22]. The technique has been used in recent years to uncover virus-host interactions at the single-cell level [23–27]. Particularly, scRNA-seq allows us to identify virus-targeted cells and compare the transcriptomic profiles between infected and bystander cells by quantifying viral and host RNA within infected cells, thereby revealing the true responses in virus-infected cells.

*Carassius auratus* herpesvirus (CaHV) is a strain of the *Cyvirus cyprinidallo2*, which was isolated from diseased gibel carp previously [11,17]. In this study, we used CaHV as a model to investigate herpesvirus-gibel carp interactions at the single-cell level using scRNA-seq. The results revealed that macrophages and mural cells are the major permissive cell types for CaHV *in vivo*. Further investigation delineated a detailed transcriptomic landscape manipulated by the virus, including pathways with pro- or antiviral functions. The reason for herpesvirus-induced hemorrhaging was also uncovered.

## Results

### Virus infection in gibel carp

**Fig 1:**
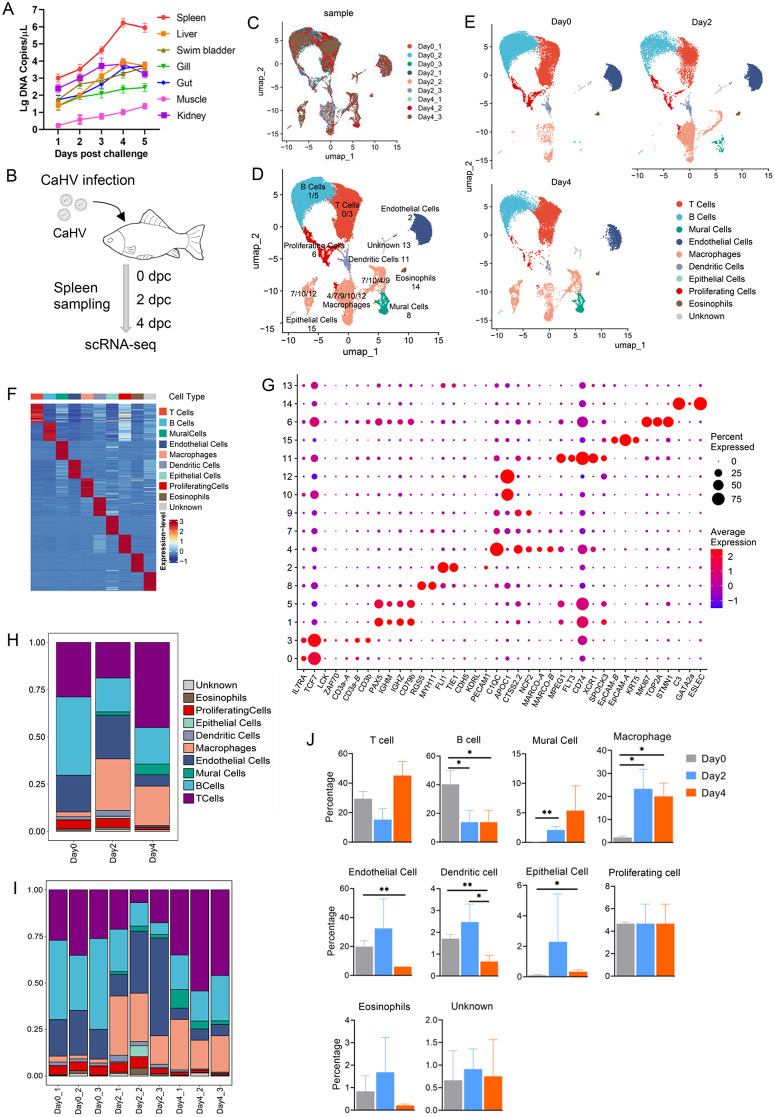
Study design and single-cell transcriptome profiling of gibel carp spleen under CaHV infection. (A) Detection of the viral DNA copy numbers in different tissues of CaHV-infected gibel carp by qPCR. (B) Study design. Single cells from the spleen of CaHV-infected gibel carp at different times post infection were sorted and subjected to scRNA-seq. The image was manually constructed. (C) UMAP visualization of cell clusters. Each dot represents a single cell and the cells from different samples are indicated with different colors. (D) and (E) Cell populations identified with marker genes in the integrated single cell data (D) or at different times post infection (E). (F) Heatmap of top30 marker genes from the clustered cell types. (G) Dot plots showing the expression levels of selected canonical cell markers in different cell clusters. Dot size indicates the percentage of cells expressing the gene in the cluster, and dot color represents the expression level. (H) and (I) Bar plots showing the proportion of the cell types at different time points (H) or at each sample (I). (J) Percentage of each cell type through the infection time obtained from the scRNA-seq data.

In our preliminary experiment and previous report [28], gibel carp challenged with CaHV typically began to die at 4–5 days post-infection (dpi). To ensure sample consistency in this study, the challenge dose that killed most gibel carp at 5 dpi was used. At 4 dpi, the challenged gibel carp exhibited symptoms of hemorrhage on skin and gills—typical characteristics of the acute hemorrhagic syndrome induced by *Cyvirus cyprinidallo2*. Subsequently, viral loads in different tissues were quantified by measuring viral genomic DNA. The results showed that the spleen exhibited the highest viral DNA levels among the examined tissues (Fig 1A), with a peak at 4 dpi.

### Overview of host transcriptome in spleen under CaHV infection by scRNA-seq

The results above have demonstrated that the spleen is a critical target organ for CaHV infection. The spleen has been considered a major peripheral lymphoid organ in fish [29]. Thus, the spleens of gibel carp at 0, 2, and 4 dpi were selected for subsequent scRNA-seq (Fig 1B). A total of 53,203 filtered cells were obtained and analyzed (S1 Table), yielding approximately 134.2 million unique transcripts. Among these cells, 21,628 (40.65%) were derived from mock-infected fish (0 dpi), 13,176 (24.77%) from fish at 2 dpi, and 18,399 (34.58%) from fish at 4 dpi. After correcting for read depth, all high-quality cells were combined into an unbatched, comparable dataset for principal component analysis (Fig 1C).

A total of 16 cell clusters were identified by analysis with graph-based clustering of uniform manifold approximation and projection (UMAP), which belonged to 10 major cell types based on classical gene markers (Fig 1D). These cell types were well-distributed across samples, while exhibiting dynamic changes in cell numbers throughout the infection period (Fig 1E). A heatmap of the top30 marker genes revealed distinct characteristics for each cell type (Fig 1F). The identified cell types included T cells (17,014 cells), B cells (14,602 cells), Mural cells (1,310 cells), Endothelial cells (8,238 cells), Macrophages (7,906 cells), Dendritic cells (867 cells), Epithelial cells (220 cells), Proliferating cells (1,789 cells), Eosinophils (335 cells), and a population of unknown cells (418 cells). As shown in Fig 1G, cluster 0 and 3 cells were identified as T cells for the co-expression of *IL7RA*, *TCF7*, and *CD3* (*CD3a-A/B* and *CD3b*) lineage marker genes. The marker genes *LCK* and *ZAP70* were also highly expressed in cluster 3 cells. Cluster 1 and 5 cells were identified as B cells based on high expression of *PAX5*, *IGHM*, *IGHZ*, and *CD79b* lineage genes. Cluster 2 cells were characterized as endothelial cells by the expression of *FLI1, TIE1,* and *PECAM1* genes. Cells in cluster 4, 7, 9, 10, and 12 constituted macrophage populations, as evidenced by the expression of lineage-specific genes *C1QC*, *APOC1*, *CTSS2.2*, *NCF2*, *MARCO-A/B*, and *MPEG1*. Detailed, the macrophages at day 0 were mainly comprised of cluster 4 cells, which highly expressed *C1QC*, *CTSS2.2*, *NCF2*, *MARCO-A/B*, and *MPEG1*. Most cells of cluster 7, 9, 10, and 12 appeared after CaHV infection (Fig 1E), in which clusters 10 and 12 possessed a high expression of *APOC1*. Cluster 6 cells were classified as a proliferating cell population based on their expression of *MKI67*, *TOP2A*, and *STMN1* lineage-specific genes. Cluster 8 cells were identified as mural cells due to the expression of the *RGS5* and *MYH11* genes. Cluster 11 cells expressed a high level of dendritic cell signature genes, including *FLT3*, *CD74*, *XCR1*, and *SPOCK3*. Interestingly, the *MPEG1* gene is also highly expressed in cluster 11, consistent with a report that *MPEG1* is highly expressed in zebrafish dendritic cells [30]. No known classical marker genes with high expression were found in cluster 13, which was designated as a population of unknown cells. Cluster 14 cells were identified as eosinophils on the basis of expression of *C3*, *GATA2a*, and *ESLEC* lineage genes. Cluster 15 cells were recognized as epithelial cells for the expression of the *EpCAM-A/B* and *KRT5* genes.

To investigate changes in cell-type abundance in the spleen during CaHV infection, the relative proportions of the 10 cell types at different time points were further analyzed using scRNA-seq data. In general, B cells, T cells, and endothelial cells were the top three most abundant cell types in the control group (Day0). However, the proportion of macrophages increased to the highest at day2 (2 dpi) and the second at day4 (4 dpi) (Fig 1H and 1I), suggesting a critical role of macrophages in the response of gibel carp to CaHV infection. Additionally, the relative abundance of B cells decreased significantly at 2 and 4 dpi. The relative percentage of endothelial cells and dendritic cells both decreased significantly at 4 dpi. In contrast, the relative proportion of mural cells increased at 2 dpi, while that of epithelial cells increased at 4 dpi (Fig 1J).

### Macrophages and mural cells are the major cell types that are infected by CaHV *in vivo*

**Fig 2:**
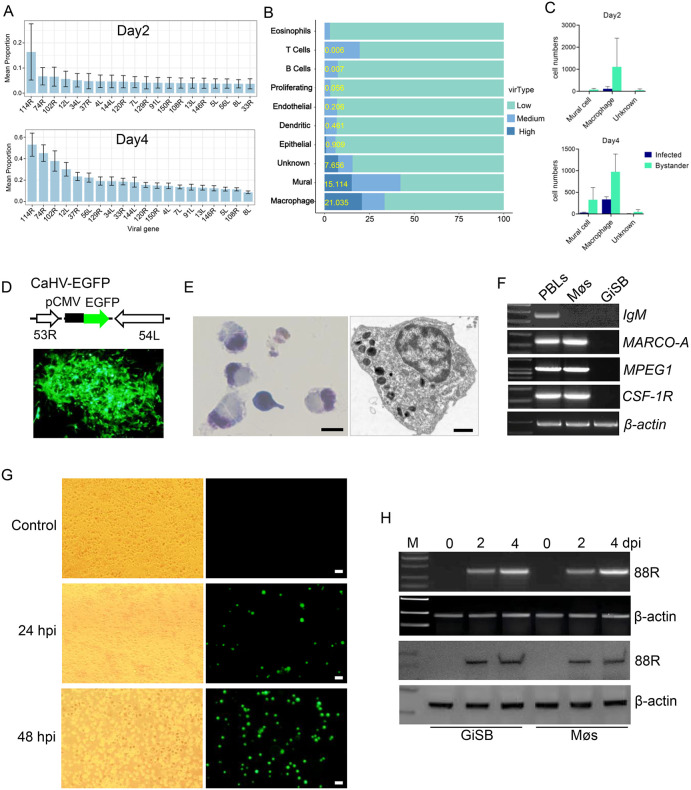
Identification of the CaHV-infected cell types. (A) Expression frequency of the top 20 viral genes at day2 or day4. The expression frequency was evaluated by the cells containing viral fragments. (B) Viral-load states in each cell type, which were defined as High, Medium, and Low. The percentage of cells with high viral load in each cell type was indicated with yellow numbers. (C) Cell numbers of infected and bystander cells in mural cells, macrophages, and the unknown group, determined using Otsu’s thresholding. (D) Construction of recombinant CaHV-EGFP virus. A pCMV-EGFP expression cassette was inserted in CaHV genome. The CaHV-EGFP-infected cells showed green signals. (E) Wright-Giemsa staining of the isolated microphages *in vitro* (left panel, bar = 20 μm) and TEM observation (right panel, bar = 1 μm). (F) Detection of the expression of representative genes in peripheral blood cells (PBLs), isolated macrophages (Møs), and GiSB cells. (G) Microscopy observation of the isolated macrophages infected by CaHV-EGFP. (H) Detection of CaHV infection of GiSB and the isolated macrophages by RT-PCR and western blot analysis of the *88R* gene expression.

After clustering the major cell types in the spleen of gibel carp, we wish to know which cell type is most likely to be infected by CaHV. The temporal expression patterns of annotated CaHV genes were first evaluated by analyzing viral unique molecular identifiers (UMIs). The proportion of viral UMIs increased from 2 dpi to 4 dpi, indicating enhanced viral replication during the infection period. The top 20 viral genes ranked by expression level were consistent between 2 dpi and 4 dpi. However, their specific ranking positions may have differed (Fig 2A). In particular, the *114R* gene showed the highest expression level from 2 dpi to 4 dpi, suggesting its potential as a diagnostic target for CaHV infection.

Following the criteria established in a previous study [23], the cells were categorized into three groups based on the percentage of viral UMIs to assess the degree of infection: low (<0.5%), medium (0.5–5%), and high (>5%). The results showed that macrophages, mural cells, and the group of unknown cells had the highest levels of viral UMIs among the cell types (Figs 2B and S1). Among cells with a high proportion of viral UMIs, macrophages accounted for 21.04%, mural cells for 15.11%, and cells in the unknown group for 7.66%. In detail, 32.61% of macrophages, 17.90% of mural cells, and 21.62% of unknown group cells clustered as “high” at 4 dpi. In contrast, only 11.47% of macrophages and 2.95% of mural cells were clustered as “high” at 2 dpi, with no unknown group cells designated as such. Thus, the primary cell types infected by CaHV *in vivo* were macrophages, mural cells, and an unknown cell type. In addition, the total number of unknown group cells was 418, with fewer than 150 observed at each detected time point.

To further investigate the infected cells while considering ambient RNA contamination, we used the Otsu’s thresholding method after logarithmic transformation based on viral UMI data to infer infected and bystander cells, as described previously [23]. The calculation yielded a threshold value, enabling the classification of each cell type into infected and bystander cells. The results showed that no infected cells were identified among mural cells and the unknown group at 2 dpi, while infected cells constituted approximately 10% of the total macrophage population (Fig 2C). At 4 dpi, infected cells were identified in all three cell types, with macrophages still containing the highest number of infected cells. These results agree with the observations from the viral UMI proportion analysis above, further confirming the major target cell types of CaHV. Furthermore, only 13 infected cells were identified in the unknown group using the Otsu’s thresholding. Given their small number and the absence of identified gene markers, the unknown group was not further investigated.

To verify the infection of macrophages and mural cells by CaHV, we constructed a recombinant CaHV containing a CMV promoter driving EGFP (CaHV-EGFP) (Fig 2D). Macrophages in gibel carp spleen were isolated by density gradient centrifugation as described previously [31]. Most isolated cells showed an eccentric nucleus, a characteristic of macrophages (Fig 2E). Expression of multiple marker genes was examined by RT-PCR. As shown in Fig 2F, the expression of *IgM*, *MARCO-A*, *MPEG1*, and *CSF-1R* can be detected in peripheral blood cells, but only *MARCO-A*, *MPEG1*, and *CSF-1R* were detected in the isolated macrophages, indicating their relatively high purity. CaHV-EGFP was used to infect the isolated macrophages. As shown in Fig 2G, cells with green fluorescence increased with infection time, indicating viral infection, which was further confirmed by detection of CaHV-*88R* (*mcp*) gene expression at mRNA and protein levels (Fig 2H).

**Fig 3:**
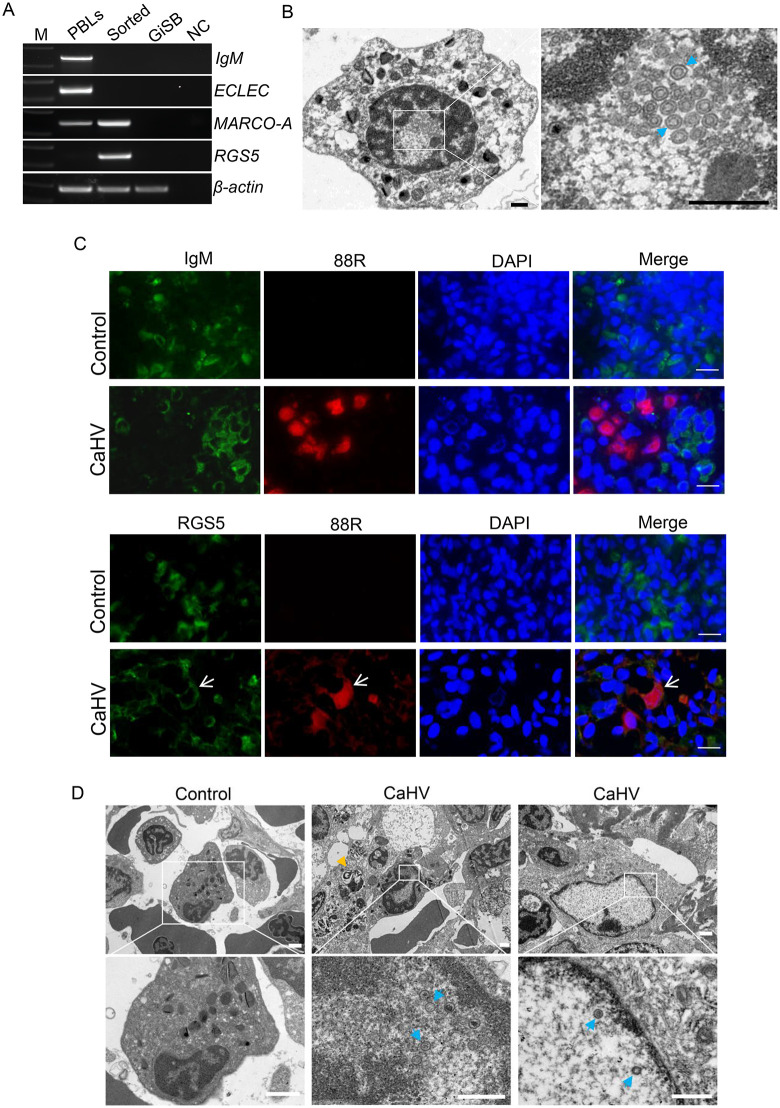
Investigation of CaHV-infected cells *in vivo.* (A) RT-PCR analysis of the expression of representative genes in CaHV-EGFP-infected cells. Splenic cells of CaHV-EGFP-infected fish were sorted by flow cytometry, and gene expression was detected. The peripheral blood cells (PBLs) and GiSB cells were used as controls. (B) TEM observation showed the virions in the nucleus of the sorted macrophages. Bar = 500 nm. (C) Immunohistochemical analysis of the spleen sections of CaHV- infected or uninfected fish. Antibodies targeting IgM, RGS5, and 88R were used to indicate B cells, mural cells, and CaHV-infected cells, respectively. Bar = 20 μm. (D) TEM analysis of the spleen sections with or without CaHV infection. The virions were indicated by blue arrows. The yellow arrow indicates the cells under programmed cell death. The scale bars of the upper and lower groups of images are 1 μm and 500 nm, respectively.

We then infected gibel carp with CaHV-EGFP and sorted spleen cells based on green fluorescence using flow cytometry. The marker genes of macrophages (*MARCO-A*) and mural cells (*RGS5*) can both be detected in the sorted EGFP-positive cells, but not the marker genes of B cells (*IgM*) and eosinophils (*ESLEC*) (Fig 3A), indicating that the infected cells contained macrophages and mural cells. TEM revealed macrophage-like cells in the sorted cells, with numerous virions in the nucleus (Fig 3B). However, although infected cells with non-macrophage morphology were present, we cannot be sure that these cells are mural cells. Then, viral infection was detected by immunohistochemistry analysis of spleen slices. As shown in Fig 3C, the anti-IgM antibody-stained cells were not colocalized with the anti-88R antibody-stained cells, indicating that B cells were not infected by CaHV. In contrast, a few anti-RGS5-labeled cells were also labeled with the anti-88R antibody, indicating that mural cells were infected with CaHV. In addition, several virions were observed in the spleen tissues by TEM observation. The cells containing virions included macrophage and non-macrophage morphologies (Fig 3D). Cells possibly under programmed cell death were also observed near the infected cells. Collectively, these results all proved that macrophages and mural cells are the major cell types infected by CaHV *in vivo*.

### CaHV-infected cells showed fewer antiviral responses (IFN) than bystander cells

**Fig 4:**
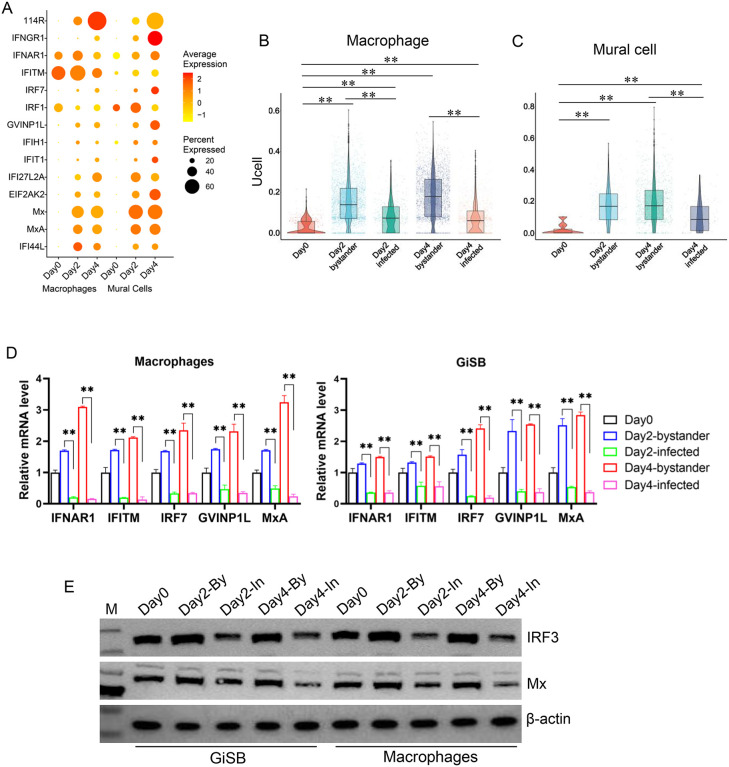
IFN responses were inhibited in CaHV-infected cells compared to bystander cells. (A) Expression level of CaHV *114R* and IFN-related genes in macrophages and mural cells at different time points. (B) and (C) Gene set score analysis of the IFN-related genes in infected and bystander cells of macrophages and mural cells. (D) RT-qPCR analysis of the expression of *IFNAR1*, *IFITM*, *IRF7*, *GVINP1L*, and *MxA* in infected and bystander cells of macrophages and GiSB cells. These cells were infected by CaHV-EGFP and sorted with flow cytometry. The cells with green fluorescence were seen as infected, and those without green signals were regarded as bystanders. (E) Western blot analysis of the expression of *IRF3* and *Mx* in cells described above.

Antiviral responses in macrophages and mural cells were evaluated by determining the relative expression of interferon (IFN)-related genes that were differentially expressed in scRNA-seq data. These genes included IFN regulatory factors (IRF1 and IRF7) and IFN-stimulated genes (ISGs). The CaHV *114R* gene was used to assess viral infection. As shown in Fig 4A, the expression level of *114R* gradually increased from day2 to day4, indicating persistent infection in the viral-permissive cells. The expression of IFN-related genes significantly increased from day0 to day2, indicating that viral infection induced a robust antiviral response. However, the extent of the increase in IFN-related gene expression from day2 to day4 did not correspond to the elevated expression level of the viral gene. Conversely, in macrophages and mural cells, the expression of several IFN-related genes on day4 was lower than or comparable to that on day2.

We further explored IFN responses in infected and bystander cells. The results revealed that, compared with day0, the IFN responses in both infected and bystander cells were enhanced on day2 or day4 (Fig 4B and 4C). Additionally, an increase in IFN responses was observed from day2 to day4 in bystander cells of macrophages. However, in both cell types, the expression levels of IFN-related genes in infected cells were significantly lower than those in bystander cells, regardless of the time point (day2 or day4). These findings suggest that CaHV can dampen innate antiviral responses in infected cells.

The infected and bystander cells from the CaHV-EGFP-infected macrophages above and the CaHV-EGFP-infected GiSB (gibel carp swim bladder) cells were separated by flow cytometry based on green fluorescence. Cells with green signals were considered infected, and those without were regarded as bystanders. Expression of several IFN-related genes was examined by RT-qPCR. As expected, the examined genes (*IFNAR1*, *IFITM*, *IRF7*, *GVINP1L*, and *MxA*) all showed significantly lower expression levels in infected cells than in bystander cells at day2 or day4 (Fig 4D). The expressions of IRF3 and Mx were further detected by western blot, for the availability of fish-specific antibodies [32,33]. As shown in Fig 4E, the protein bands of IRF3 and Mx in infected cells were both weaker than those in bystander cells. The situations are similar in primary-cultured macrophages and passage-cultured GiSB cells, confirming the inhibition of the innate antiviral responses by CaHV in different cell types.

### Macrophage responses during CaHV infection

We further investigated virus-induced responses in sensitive cell types by analyzing differentially expressed genes (DEGs). For each cell type, DEGs can be categorized into two types: one derived from comparisons of cells at day2 or day4 with control cells (day0), and the other from comparisons of infected cells with bystander cells.

**Fig 5:**
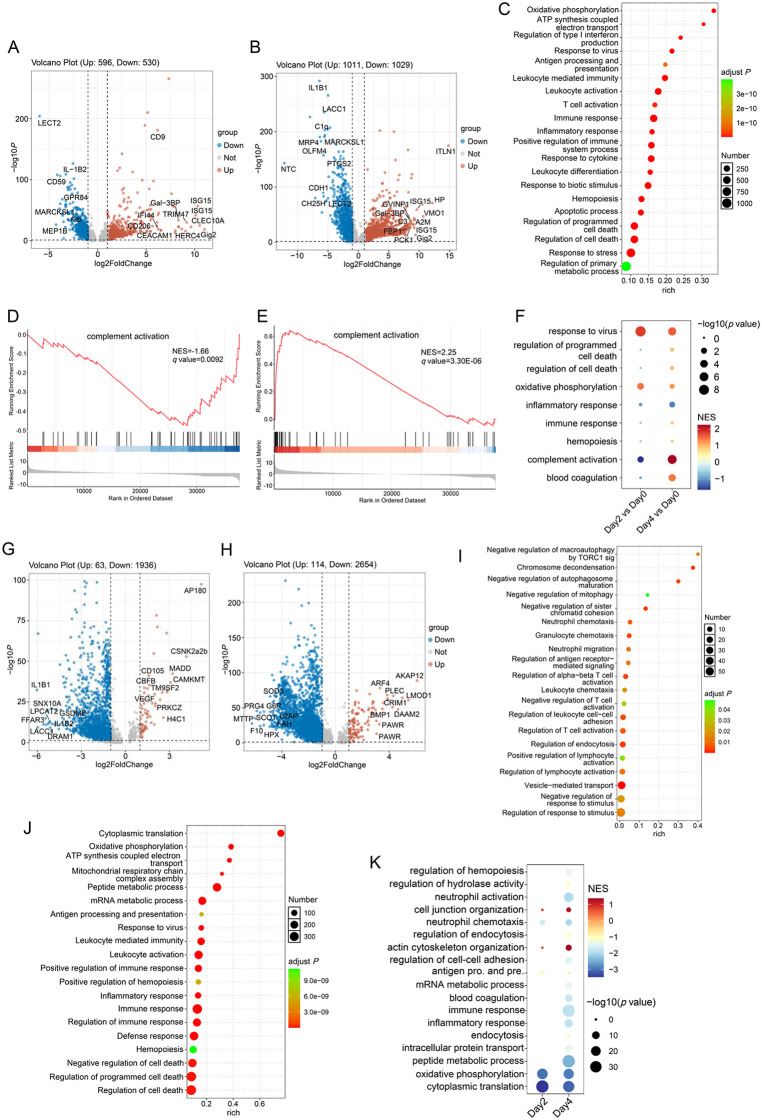
Macrophage responses in the spleen of CaHV-infected gibel carp. (A and B) Volcano plot of the DEGs of macrophages in day2 vs day0 (A) and day4 vs day0 (B) groups. (C) GO terms enrichment of the DEGs in the day2 vs day0 group. (D and E) GSEA analysis of the GO term complement activation in the day2 vs day0 group (D) and the day4 vs day0 group (E). (F) Comparison of the GSEA results of representative GO terms between day2 vs day0 and day4 vs day0 groups. (G and H) Volcano plot of the DEGs of infected vs bystander group at 2 dpi (G) and 4 dpi (H). (I and J) GO terms enrichment of the upregulated DEGs (I) or downregulated DEGs (J) of infected vs bystander group at 2 dpi. (K) Comparison of the GSEA results of representative GO terms of the infected vs bystander groups at day2 and day4.

Several ISGs were present among the top upregulated DEGs in the day2 vs day0 group (Fig 5A), including *Gig2* (*grass carp reovirus (GCRV)-induced gene 2*)—the most significantly upregulated DEG, which has been identified as a fish-specific ISG with antiviral activity [34] —as well as *ISG15*, *IFI44*, etc. Additional immune response-related genes were also observed, including those encoding C-type lectin domain family 10 member A (CLEC10A, also known as macrophage galactose-type lectin) and a homologue of galectin-3-binding protein (Gal-3 BP). *CLEC10A* has been detected on tolerogenic antigen-presenting cells (APCs), including M2a macrophages [35], and exhibits immune regulatory functions. *Gal-3 BP*, by contrast, is recognized as a soluble marker of M1 macrophages and is upregulated in multiple viral infections, where it activates antiviral innate immune responses [36,37]. Other top-upregulated DEGs include *TRIM47* (*tripartite motif-containing protein 47*), which has been reported to be highly expressed in macrophages and to interact with MAVS to enhance IFN production [38]. In addition, classical macrophage markers *CD9* and *CD206* (*MRC1*) were also among the top upregulated DEGs.

Although the ISGs *Gig2* and *ISG15* remained among the top 30 upregulated DEGs in the day4 vs day0 group, genes associated with macrophage generation/differentiation/phagocytosis and glucose metabolism were significantly upregulated in the group (Fig 5B). The most highly upregulated gene, *ITLN1*, encodes an intelectin-1a (ITLN)-like protein, which can enhance macrophage phagocytosis [39]. The second most upregulated gene, *HP*, encodes haptoglobin, a protein reported to modulate macrophage polarization toward the M2 phenotype [40]. Another highly upregulated gene, *VMO1*, encodes a vitellogenin-like protein that also possesses phagocytosis-enhancing activity [41]. Alpha-2-macroglobulin (*A2M*) exhibits abilities such as enhancing immune cell migration and proliferation, and facilitating antigen presentation [42]. Phosphoenolpyruvate carboxykinase (PCK1) and fructose-1,6-bisphosphatase 1 (FBP1) are key enzymes in gluconeogenesis. In addition, the Complement C3-like encoding gene *C3* was significantly upregulated.

The top downregulated DEGs in the day2 vs day0 group included *LECT2* (*leukocyte cell-derived chemotaxin-2-like*) and *TOB1*—both reported as regulators of innate immune responses [43,44]—as well as *IL-1β2* and *GPR84*, which have pro-inflammatory functions [45]. Others included genes encoding coagulation factor Ⅲb (*f3b*) and CD59, which could protect cells from the complement system [46]. Genes with pro-inflammatory activity were also found among the top downregulated DEGs in the day4 vs day0 group, such as *NTC* (*galactose-specific lectin nattectin-like*) and *CH25H* (*Cholesterol 25-hydroxylase*) [47,48], along with immune regulators like *LACC1* (*Laccase domain-containing protein 1*) [49]. There were also several DEGs downregulated in both day2 and day4 groups.

As indicated by the top regulated DEGs, enrichment analysis revealed that DEGs in the day2 vs day0 group were significantly enriched in pathways related to immune response, inflammatory response, cell death, and hemopoiesis. Additionally, the oxidative phosphorylation pathway was significantly enriched (Fig 5C). In the day4 vs day0 group, in addition to the aforementioned terms, GO terms related to complement activation and blood coagulation were significantly enriched (S2A Fig). Gene Set Enrichment Analysis (GSEA) was further performed to assess the gene expression in the enriched GO terms. As shown in Fig 5D and 5E, the GO term complement activation was significantly enriched and downregulated in the day2 vs day0 group, but upregulated in the day4 vs day0 group. A comparison of the GSEA results of representative GO terms between day2 vs day0 and day4 vs day0 groups was shown in Fig 5F. The significantly activated and upregulated GO terms also included response to virus and oxidative phosphorylation at the day2 vs day0 group, and response to virus and blood coagulation at the day4 vs day0 group. Simultaneously, the GO term inflammatory response was significantly downregulated in the day4 vs day0 group. Other GO terms, such as regulation of cell death and immune response, showed a slight upregulation at day4 vs day0 group, but this was not significant.

A total of 1,999 DEGs (63 upregulated) and 2,768 DEGs (114 upregulated) were identified in the infected vs bystander groups at day2 and day4, respectively. Marked differences were observed between the DEGs of the infected vs bystander group and those of the day2/day4 vs day0 group. No upregulated IRFs or ISGs were detected in the infected vs bystander group (Fig 5G and 5H), which is consistent with the aforementioned results. Enrichment analysis showed that at day2, DEGs related to vesicle-mediated transport, endocytosis, neutrophil chemotaxis, T cell activation, and cell-cell adhesion were significantly upregulated in infected cells. In contrast, those related to translation, oxidative phosphorylation, metabolic process, and immune/inflammatory response were significantly downregulated (Fig 5I and 5J). At day4, DEGs associated with actin cytoskeleton organization, cell junction organization, transport, and hydrolase activity were significantly upregulated, whereas DEGs related to translation, metabolic process, and immune response were still significantly downregulated (S2B and S2C Fig). In addition, DEGs involved in the regulation of programmed cell death, antigen processing and presentation, hemopoiesis, and blood coagulation were also significantly downregulated. Further GSEA analysis showed a result similar to the GO enrichment above. Activities of most GO terms, including immune and metabolic-related terms, showed significant downregulation at day4 (Fig 5K).

The top DEGs were consistent with the enrichment analysis. For example, the most upregulated gene at day2 (Fig 5G), *AP180*, has been reported to be involved in clathrin-mediated endocytosis [50]. The most downregulated gene, *IL1B1*, encodes a pro-inflammatory cytokine [51]. At day4 (Fig 5H), the most upregulated gene in infected cells was *AKAP12* (*A-kinase anchor protein 12*), whose product has been identified as a scaffold protein that mediates signaling and even regulates mRNA localization and translation [52,53]. Two other DEGs, *DAAM2* and *PLEC*, have been reported to participate in cytoskeleton organization. The top downregulated DEGs included those involved in lipid metabolism (*MTTP*), glycometabolism (*G6P*), ketone body catabolism (*SCOT*), and tyrosine catabolism (*FAH*). The blood coagulation factor-encoding gene *F10* was also among the top-downregulated DEGs.

### Subpopulations of macrophages and their responses during CaHV infection

**Fig 6:**
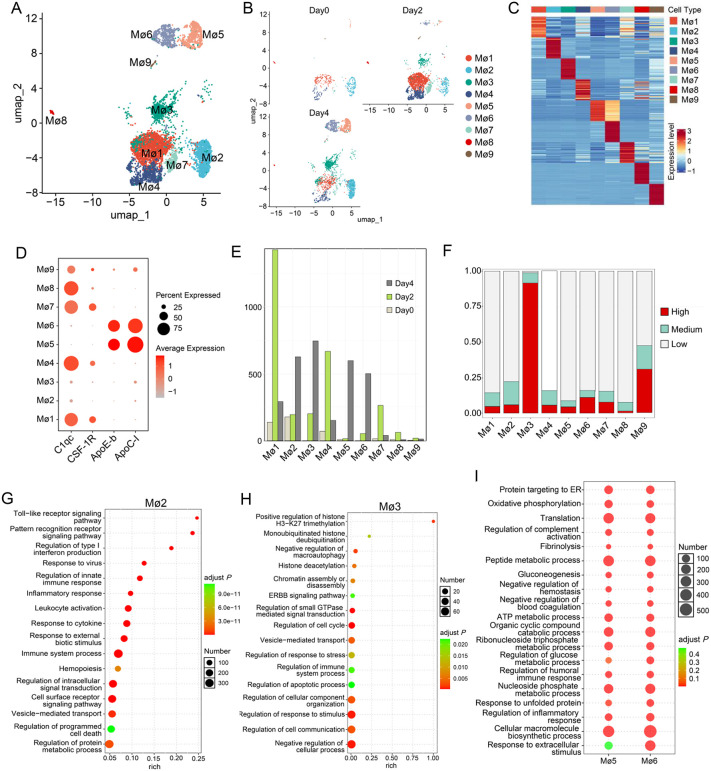
Characterization of the subpopulations of macrophages and their responses to CaHV infection. (A) and (B) UMAP overview of the subpopulations of macrophages in the integrated scRNA-seq data (A) or at different time points (B). (C) Heatmap of expressed genes of the nine macrophage subpopulations. (D) Expression levels of *C1qc*, *CSF-1R*, *ApoE-b*, and *ApoC-I* in the nine macrophage subpopulations. (E) Cell numbers of the nine macrophage subpopulations at different time points. (F) Proportions of the cells containing varying degrees of viral UMIs (High, Medium, and Low) in the nine macrophage subpopulations. (G, H, and I) Representative GO terms enriched in the highly expressed genes of Mø2 (G), Mø3 (H), and Mø5/Mø6 (I).

The above results showed that macrophages are the most sensitive target cells for CaHV. To investigate whether distinct macrophage subtypes emerge during infection and the corresponding responses, macrophage classification was conducted. This analysis yielded nine subset clusters (designated as Mø0–Mø9) with dynamic changes in cell numbers across different infection time points (Fig 6A and 6B). The top-ranked cluster-signature genes for each subset are shown in Fig 6C, revealing distinct gene expression profiles among the nine subpopulations, although some genes show similar expression patterns across subsets. Four signature genes (*C1qc*, *CSF-1R*, *ApoE-b*, and *ApoC-I*) were identified for these nine subsets. *C1qc* has been considered a marker of a subpopulation of tissue-resident macrophages [54], while *CSF-1R* is closely associated with macrophage differentiation and tissue richness [55]. *ApoE-b* and *ApoC1* are highly expressed in some macrophage subpopulations and possess anti-inflammatory functions [56,57]. *C1qc* was highly expressed in the Mø1, Mø4, Mø7, and Mø8 subsets, with moderate expression in the Mø9 subset. *CSF-1R* exhibited moderate expression in the Mø1, Mø4, and Mø7 subsets. Both *ApoE-b* and *ApoC-I* were highly expressed in the Mø5 and Mø6 subsets, whereas low expression levels of these four genes were detected in the Mø2 and Mø3 subsets (Fig 6D).

Notably, not all subset clusters were present in the day0 samples. The Mø3 and Mø6 subpopulations were absent on day0, indicating that seven macrophage subsets exist in the spleen of healthy gibel carp. The proportions of these macrophage subsets changed dynamically as the infection progressed. On day0, Mø2 (accounting for ~41.81% of total macrophages) and Mø1 (~32.78%) were the two most abundant subpopulations. The proportion of Mø1 increased to about 49.1%, while that of Mø2 decreased to ~6.7% on day 2. Meanwhile, two novel subsets (Mø3, ~ 6.9%; Mø6, ~ 1.8%) emerged, which were attributed to CaHV infection. Additionally, the proportion of Mø5 decreased from ~2.1% (day0) to ~0.5% (day2). On day4, the proportion of Mø3 increased to ~25.1%, making it the most abundant subset at this time point. The proportions of Mø2 (~21.1%), Mø5 (~20.1%), and Mø6 (~16.8%) all showed an obvious increase from day2 to day4, whereas the proportion of Mø1 decreased sharply during the same period (Figs 6E, S3A, and S2 Table).

To assess the infection status of each macrophage subset, cells within each subset were categorized as high, medium, or low based on viral UMIs, as described above. At 2 dpi, subsets with high infection levels (>5%) included Mø2 (5.67%), Mø3 (86%), Mø5 (100%), Mø6 (90.57%), and Mø9 (26.31%). At 4 dpi, the high-infection subsets were Mø1 (27.59%), Mø2 (7.22%), Mø3 (93.12%), Mø4 (17.88%), Mø7 (56.41%), and Mø9 (46.15%) (Figs 6F and S3 and S4 Table). It is important to note that the high-viral-fragment-containing subsets (Mø3 and Mø6) were not present on day 0. Specifically, the majority of Mø3 cells were classified as “high” at both 2 and 4 dpi, and the proportion of Mø3 became the highest at 4 dpi. These findings suggest that CaHV infection alters the gene expression profiles of target cells and induces the generation of two novel subsets (Mø3 and Mø6), with Mø3 accumulating the majority of infected cells.

The highly expressed genes of these subsets were analyzed by GO enrichment analysis, which revealed distinct characteristics for several subsets. As shown in Fig 6G, the immune-related terms, including pattern recognition receptor signaling pathway, regulation of type I interferon production, inflammatory response, and immune system process, were significantly enriched in Mø2. For Mø3, several significantly enriched GO terms focused on epigenetic regulation, including histone trimethylation/deubiquitination/deacetylation, and chromatin assembly or disassembly (Fig 6H). The two subsets, Mø5 and Mø6, showed similar enriched GO terms, including several metabolic processes such as oxidative phosphorylation, gluconeogenesis, and peptide metabolic process, as well as immune- and inflammatory-related responses (Fig 6I). The significantly enriched GO terms of these subsets corresponded to their infection status.

An important function of macrophages is antigen presentation, which requires the involvement of major histocompatibility complex (MHC) molecules. Therefore, the expression levels of MHC-related genes in the nine subsets were examined across the infection period. Notably, multiple genes with identical names may exist, which is a consequence of the amphitriploid genome of gibel carp [9]. As shown in S3B Fig, Mø1, Mø2, and Mø4 exhibited relatively high expression of MHC-related genes on day0, whereas most MHC-related genes showed low expression in Mø5 and Mø9 at this time point. By day2, the expression levels of MHC-related genes increased—including an obvious upregulation in Mø5 and Mø9—indicating a response to CaHV infection. The two newly emerged subsets (Mø3 and Mø6) at day 2 both displayed high expression levels of MHC-related genes. On day 4, most subsets (including Mø3) maintained high expression of these genes; however, expression levels decreased in Mø6, along with a decrease in viral infections in that subset, and variable expression patterns were observed in some other subsets.

### CaHV infection induced macrophage differentiation

**Fig 7:**
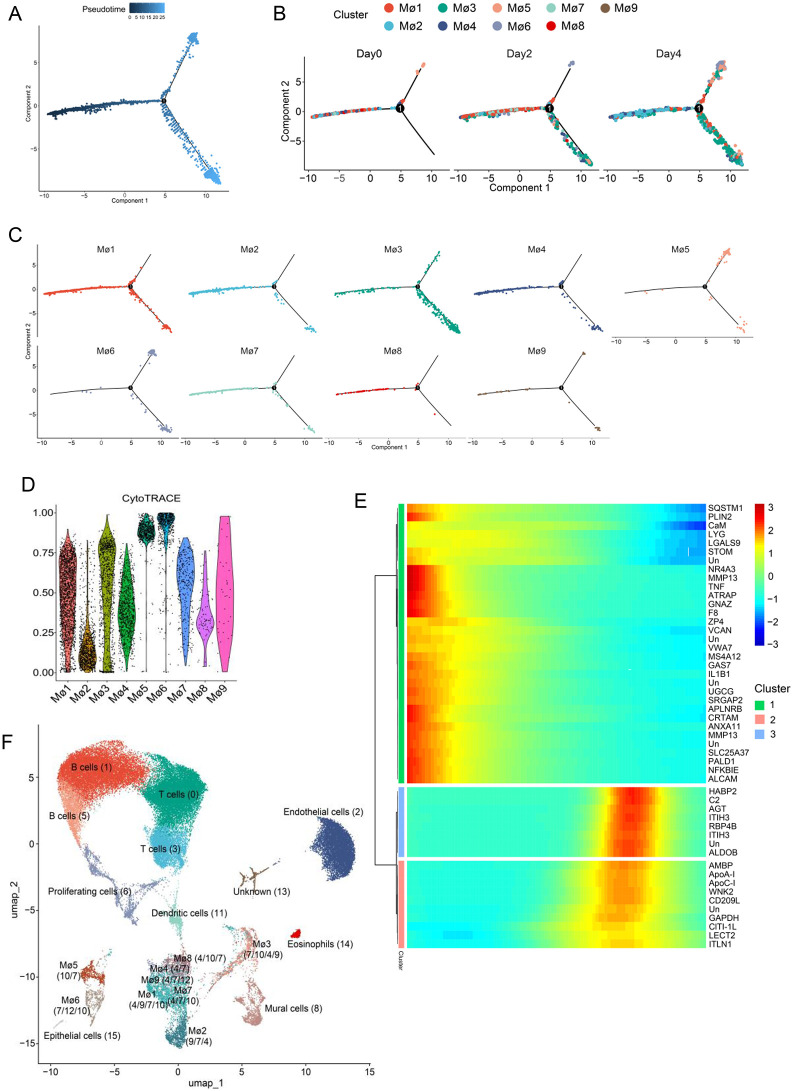
Characterization of the putative differentiation relationship among the macrophage subpopulations. (A) Pseudotime trajectory of macrophages constructed by Monocle2. The gradient from dark to light blue indicates the pseudotime ordering. (B and C) Distribution of macrophages in the trajectory at different time points (B) or in different subpopulations (C). (D) Stemness score analysis of the nine macrophage subpopulations. (E) Expression heatmap of significant marker genes revealed by the cell trajectory analysis. (F) Correspondence between the nine macrophage subpopulations (Mø1- Mø9) and the originally identified cell clusters (0-15) in UMAP analysis. The cell clusters with cell numbers more than 5 were listed in brackets, and those with larger quantities were placed first.

To investigate the potential associations among macrophage subpopulations, pseudotemporal analysis was performed using Monocle2. As shown in Fig 7A, the macrophages were categorized into three pseudotime trajectory states, with two of these states identified in samples of day0 (Fig 7B). On day0, the clusters of Mø1, 2, 4, 7, 8, and 9 were localized in a state different from the Mø5 cluster. The third state appeared on day 2 and day 4, possibly related to CaHV infection, which included all of the nine subsets. Interestingly, Mø3 and Mø6, which represent virus-infected cells, were not only aggregated in the third state but also distributed in the other two states (Fig 7C), although Mø6 was mainly located in a terminally differentiated state. Thus, the distribution of the nine subsets across the three states suggests a close relationship among them, which was influenced by CaHV infection.

To further explore the relationship between the day0 subsets and the two subsets that emerged following virus infection, the differentiation potential of the nine subsets was evaluated using CytoTRACE. The results showed that the Mø6 subset was predicted to be in a differentiated state similar to that of Mø5; meanwhile, Mø3 was located in an intermediate state (S4A Fig). Further stemness score analysis revealed that Mø1 and Mø3 exhibited similar scores, whereas Mø5 and Mø6 shared comparable scores (Fig 7D). Given the variation in cell numbers, we hypothesize that most Mø3 cells are derived from the CaHV-infected Mø1 cells, and that Mø6 cells may originate from Mø5 cells.

Cell trajectory branching analysis also identified the top genes of three groups (Fig 7E). The top genes in Cluster 2 include *AMBP* (Alpha-1-microglobulin bikunin precursor), *ApoA-I*, *ApoC-I*, *CD209L*, *GAPDH*, *LECT2*, and *ITLN1*. *ApoA-I*, *ApoC-I*, and *ITLN1* have been reported to possess anti-inflammatory functions [57–59]. CD209 is highly expressed on monocyte-derived M2 macrophages and DCs [60]. The expression levels of these genes were extremely low on day 2 but increased on day 4 in these macrophage subpopulations (S5 Table). These findings demonstrate that CaHV infection induces infected macrophages to adopt an anti-inflammatory state (M2 type). This conclusion is further supported by the observation that several pro-inflammatory genes were among the top downregulated DEGs in the day2/day4 vs day0 comparison groups.

We further analyzed the correspondence between the 9 Mø subsets and the 5 cell clusters (4/7/9/10/12; Fig 1G) obtained in the original cell clustering via UMAP (Figs 7F and S4B). The results showed that cells of cluster 4 were present in all 9 Mø subsets, accounting for the majority of Mø1/4/7/8. Cluster 7 did not appear in day0, indicating its generation was induced by CaHV infection. Cells of the cluster were also found in all Mø subsets, with the highest proportion (90.12%) in Mø3. Cluster 9 can be found at the three time points and in Mø1/2/3/4/7/9, where it occupied 90.43% of the Mø2 cells. Cluster 10 was mainly observed in Mø5 at day4, comprising more than 99% of the cells in the subset at this time point. Cluster 12 was another cell population that was not found at day0 and mainly appeared in Mø6, accounting for 92.37% of Mø6 cells at day4. It can be observed that cluster 4 and cluster 9 constitute the Mø subsets at day0, except Mø5, which contains cluster 10. After virus infection, clusters 7 and 12 appeared and formed Mø3 and Mø6, respectively.

### Mural cell responses during CaHV infection

**Fig 8:**
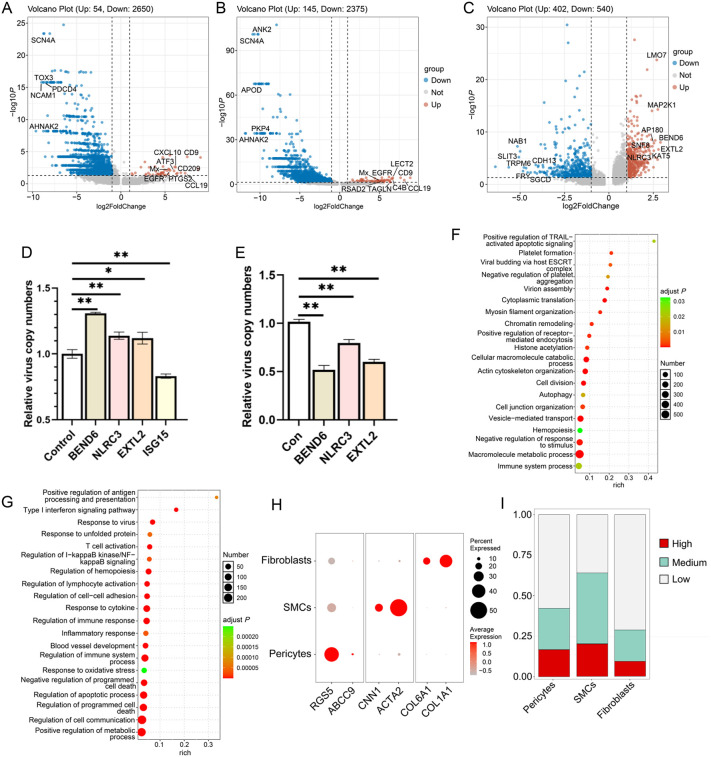
Mural cell responses under CaHV infection. (A and B) Volcano plot of the DEGs of mural cells of day2 vs day0 (A) and day4 vs day0 (B) groups. (C) Volcano plot of the DEGs of the infected vs bystander group at 4 dpi. (D) Relative virus copy numbers in GiSB cells overexpression of *BEND6*, *NLRC3*, *EXTL2*, or *ISG15* and infected with CaHV. (E) Relative virus copy numbers in GiSB cells that were transfected with plasmids expressing shRNA targeting *BEND6*, *NLRC3*, or *EXTL2*, and then infected with CaHV. (F and G) GO terms enrichment of the upregulated DEGs (F) or downregulated DEGs (G) of the infected vs bystander group. (H) Mural cells were clustered into three subpopulations, and the related marker gene expression levels were analyzed. (I) Proportions of the cells containing varying degrees of viral UMIs (High, Medium, and Low) in the mural cell subpopulations.

For mural cells of day2 vs day0 group, 54 up-DEGs and 2650 down-DEGs were identified (Fig 8A). The top up-DEGs include *CD9*, *CCL19*, *CD209*, *PTGS2*, *CXCL10*, *ATF3*, *EGFR*, and *Mx*. The top down-DEGs include *AHNAK2*, *NCAM1*, *TOX3*, *SCN4A*, and *PDCD4*. Enrichment analysis showed that the up-DEGs were significantly enriched in GO terms related to prostaglandin biosynthesis, vasoconstriction, response to virus, response to cytokine, positive regulation of immune response, positive regulation of cell adhesion, and blood vessel morphogenesis (S5A Fig). The significantly enriched GO terms for the down-DEGs include cytoplasmic translation, autophagy, lymphocyte activation, hemopoiesis, immune system process, regulation of programmed cell death, and so on (S5B Fig).

In the day4 vs day0 group, 145 up-DEGs and 2375 down-DEGs were identified (Fig 8B). The top up-DEGs contain *CCL19*, *CD9*, *LECT2*, *C4B*, *TAGLN*, *EGFR*, *RSAD2*, and *Mx*, while the top down-DEGs include *AHNAK2*, *SCN4A*, *APOD*, *ANK2*, and *PKP4*. Compared with the enriched GO terms of the up-DEGs in the day2 vs day0 group, new GO terms related to nitric oxide-mediated signaling, type I interferon signaling pathway, plasminogen activation, negative regulation of blood coagulation, and negative regulation of programmed cell death were added in the day4 vs day0 group. For the down-DEGs, the GO terms related to translation, immune response, and hemopoiesis remained enriched on day4. Meanwhile, GO terms related to interleukin and positive regulation of programmed cell death emerged at this time point (S6 Fig). Enrichment analyses at both time points indicated an inhibition of immune response and programmed cell death.

Using the OTSU value described above to distinguish infected and bystander cells, no infected cells were identified in mural cells on day 2, suggesting that the infection level was relatively mild at this time point. A total of 942 DEGs (402 upregulated) were identified in the infected vs bystander group on day 4. The top up-DEGs include *BEND6*, *EXTL2*, *MAP2K1*, *LMO7*, *AP180*, *KAT5*, *SNF8*, and *NLRC3*, while the top down-DEGs contain *NAB1*, *SLIT3*, *TRPM6*, *FRY*, *SGCD*, and *CDH13* (Fig 8C). It has been reported that *BEND6* and *NLRC3* act as negative regulatory factors in innate immunity [61,62]. We then overexpressed *BEND6*, *NLRC3*, *EXTL2*, and *ISG15* in GiSB cells and infected these cells with CaHV. qPCR analysis showed that the viral copy numbers significantly increased in cells expressing *BEND6*, *NLRC3*, and *EXTL2*, while decreasing in cells expressing *ISG15*—a well-known antiviral ISG (Fig 8D). Furthermore, the expression of *BEND6*, *NLRC3*, and *EXTL2* was suppressed by plasmid-expressed shRNAs. Detection of viral copy numbers showed that knockdown of the three genes significantly inhibited virus infection (Fig 8E). These data indicated the important roles of *BEND6*, *NLRC3*, and *EXTL2* in CaHV infection.

Enrichment analysis showed that the up-DEGs were enriched significantly in GO terms such as positive regulation of TRAIL-activated apoptotic signaling pathway, autophagy, viral budding via host ESCRT complex, platelet formation, negative regulation of platelet aggregation, and so on (Fig 8F). The GO terms for the down-DEGs were enriched in immune response, such as positive regulation of antigen processing and presentation, type I interferon signaling pathway, and T cell activation (Fig 8G). In addition, cell death-related terms, such as regulation of programmed cell death and regulation of apoptotic process, were significantly enriched.

We also explored distinct subtypes of mural cells. As shown in Figs 8H and S7A, the mural cells could be further classified into fibroblasts, smooth muscle cells (SMCs), and pericytes based on gene markers. The numbers of the three subsets all increased with increasing infection time (S7B Fig). Viral UMIs analysis showed that CaHV could infect all three cell types. The cells with “High” viral UMIs can be identified in all three cell types (Fig 8I), although fibroblasts contained the fewest “High” cells, suggesting the complexity of CaHV-permissive cells.

### Heparan sulfate and ESCRT complex are crucial for CaHV infection

**Fig 9:**
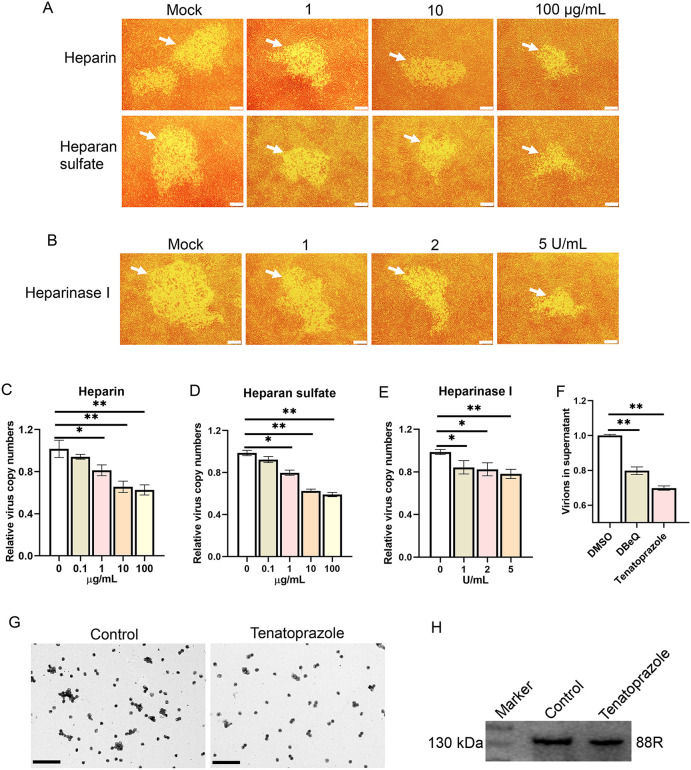
Evaluation of the roles of heparan sulfate (HS) and ESCRT complex on CaHV infection. (A) Heparin or HS treatment reduced the CPE caused by CaHV. GaHV were pretreated with different concentrations of heparin or HS and then infected GiSB cells. The CPE (indicated by white arrows) was observed at 5 days post infection (dpi). Bar = 50 μm. (B) Heparinase I treatment reduced the CPE caused by CaHV. GiSB cells were pretreated with different concentrations of heparinase I and then infected with CaHV. The CPE was observed as above. Bar = 50 μm. (C-E) Detection of the CaHV copy numbers in samples of virus binding assay by qPCR analysis. (F-H) DBeQ and tenatoprazole treatment reduced the virions in cell culture supernatant. Virion numbers in the supernatant were detected by qPCR analysis (F) or by TEM observation of the negatively stained virions (G). Bar = 1 μm. Virion proteins were also detected by western blot analysis of the 88R protein (H).

The results above demonstrate that *EXTL2* may participate in CaHV infection. Since EXTL2 is one of the key enzymes involved in the biosynthesis of heparan sulfate (HS) [63], which is a type of cell-surface glycosaminoglycan that can be utilized by viruses to facilitate infection [64], we investigated the role of HS in CaHV infection. Reduced CPE was observed in cells infected with the CaHV pretreated with different concentrations of heparin (a structural homolog of sulfated HS) or HS (Fig 9A), or in CaHV-infected cells that were pretreated with heparinase I, which could remove cell surface HS (Fig 9B), indicating the important roles of HS in CaHV infection. Subsequently, a virus-cell binding assay was performed using CaHV pretreated with heparin or HS. As shown in Fig 9C and 9D, the binding of CaHV was inhibited with increasing concentrations of heparin and HS. A reduction of about 40% compared to the control was observed at 10 μg/mL in both assays. In cells pretreated with heparinase I, the virus-cell binding assay also showed that heparinase I treatment decreased CaHV binding (Fig 9E). Collectively, these results confirm that HS could be an important binding factor for CaHV.

The enrichment analysis above showed that the GO term “viral budding via host ESCRT complex” was significantly enriched among the up-DEGs. To investigate the roles of host ESCRT (endosomal sorting complex required for transport) complex in CaHV infection, we treated CaHV-infected GiSB cells with two ESCRT activity inhibitors, DBeQ and tenatoprazole [65,66], and then quantified virions in the cell supernatants. The qPCR results showed that both DBeQ and tenatoprazole treatments significantly reduced the virion copy numbers in cell supernatants compared to the control (Fig 9F). Electron microscopy of negatively stained virions and western blot analysis of the viral major capsid protein in tenatoprazole-treated samples showed similar results (Fig 9G and 9H). Thus, the results indicated that the ESCRT complex could be involved in the late stage of CaHV infection.

### Endothelial cell is one of the targets for virus-induced hemorrhage

**Fig 10:**
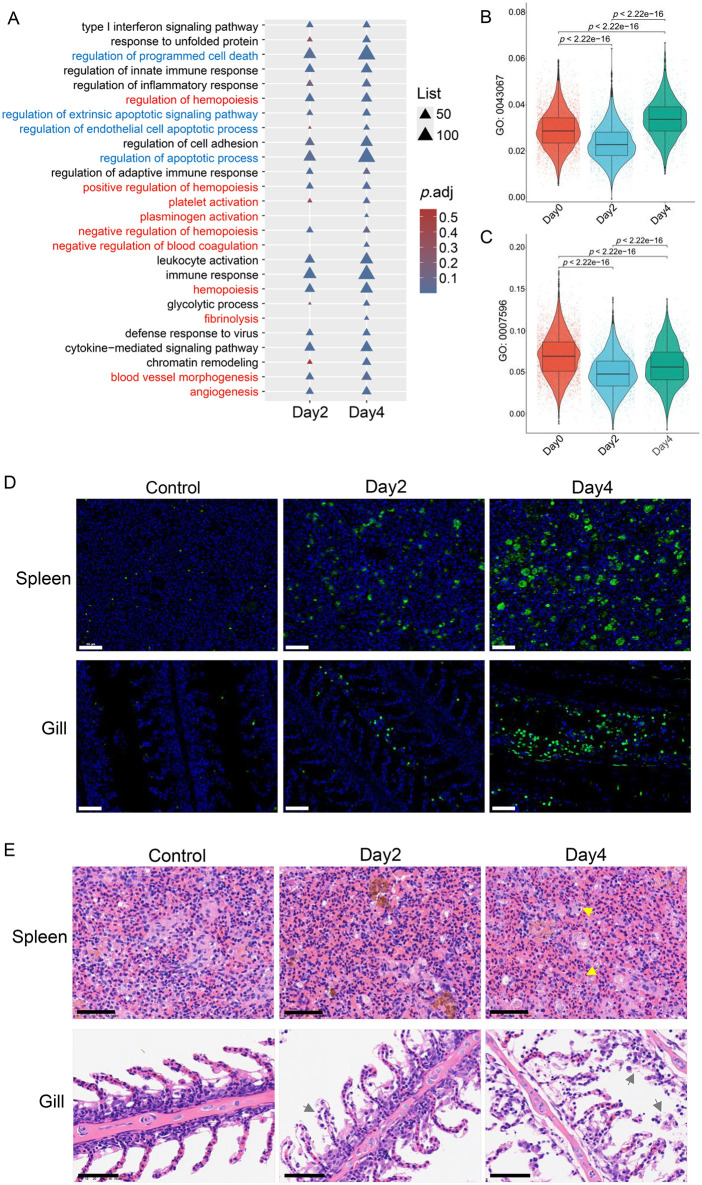
Responses of endothelial cells to CaHV infection and virus induced pathology. (A) Enriched GO terms of DEGs of endothelial cells in day2 vs day0 or day4 vs day0 groups. Blue font indicates cell death-related GO terms. Red font indicates hemopoiesis and blood coagulation related GO terms. (B) and (C) Gene set score analysis of the DEGs involved in two GO terms: regulation of programmed cell death (GO:0043067) and blood coagulation (GO:0007596). (D) Apoptosis analysis of the cells of the spleen and gill through CaHV infection by TUNEL staining. Apoptosis-positive cells were indicated by green color. Bar = 50 μm. (E) Pathology of spleen and gill through CaHV infection observed by HE staining. Yellow and gray arrows indicate the damaged ellipsoids and gill filaments, respectively. Bar = 50 μm.

A typical feature of gibel carp infected with herpesvirus is hemorrhage, indicating damage to the circulatory system. In addition to mural cells, endothelial cells constitute another major component of blood vessels, and this cell type was identified in the present study. Analysis of cell percentage data revealed that the proportion of mural cells increased with the duration of infection (Fig 1J). By contrast, the percentage of endothelial cells decreased significantly at 4 dpi. We then analyzed the DEGs of endothelial cells at different time points. Beyond GO terms related to immune response (e.g., type I interferon signaling, innate immune response, inflammatory response), the significantly enriched GO terms also contained those associated with cell death, such as regulation of programmed cell death, regulation of extrinsic apoptotic signaling pathway, and regulation of endothelial cell apoptotic process (Fig 10A). The number of DEGs enriched in these cell death-related pathways increased from day2 to day4, suggesting a gradual enhancement of such responses. Simultaneously, GO terms related to hematopoiesis and the blood system (regulation of hemopoiesis, angiogenesis, etc) were significantly enriched, with their enrichment levels increasing over the infection period, indicating the host responses to the damaged circulatory system. Moreover, the coagulation-related GO term “platelet activation” was significantly enriched at day4, indicating a host response to hemorrhage. However, GO terms including plasminogen activation, negative regulation of blood coagulation, and fibrinolysis were also enriched at day4, which have anticoagulation functions and may thereby contribute to hemorrhage.

Gene set score analysis was further performed to evaluate the activities of two representative GO terms: regulation of programmed cell death (GO:0043067) and blood coagulation (GO:0007596). The results showed that the activities of the GO term “regulation of programmed cell death” decreased significantly at day2 compared to day0, but increased remarkably at day4 (Fig 10B), indicating intense cell death during the late stage of infection. The activities of the “blood coagulation” showed a significant decline at both day2 and day4 compared to day0 (Fig 10C), although there was an increase from day2 to day4, indicating an impairment of coagulation function during virus infection.

Apoptosis represents a key pathway of programmed cell death. We examined apoptosis in the spleen and gill via TUNEL staining of tissue sections. Gill tissue is responsible for oxygen exchange and contains abundant capillaries. The results showed that the TUNEL-positive signals (green) in both tissues increased with infection time (Fig 10D). Hematoxylin-eosin (H&E) staining also showed progressive pathological changes in these two tissues over the infection period, including enlargement of the red pulp and damaged ellipsoids in the spleen, as well as cell destruction in gill filaments (Fig 10E). Importantly, the damage to ellipsoids and collapse of gill filaments were consistent with the increased apoptotic activity. Thus, the activated programmed cell death of endothelial cells, reduced blood coagulation capacity, and abnormal activation of anticoagulant pathways could collectively account for the hemorrhaging induced by CaHV.

## Discussion

CaHV, or other isolates of the *Cyvirus cyprinidallo2*, are major pathogens of cultured gibel carp and other related species and have caused devastating losses on aquaculture farms across several Asian countries. Once the disease breaks out, it progresses rapidly with high fish mortality, and typical signs such as gill hemorrhage emerge. Although multiple strains of *Cyvirus cyprinidallo2* have been reported, critical knowledge gaps remain, including the *in vivo* target cells of the virus, its mechanisms for modulating host responses, and the molecular basis of pathogenesis. These gaps continue to impede the development of effective antiviral strategies. Using the CaHV strain as a model, the present study employed scRNA-seq to dissect the host’s refined splenic transcriptomic responses and viral gene expressions during infection. Combined with experimental validation, we identified, for the first time, the principal splenic cell types targeted by CaHV, mapped the host genes and pathways it manipulates, and provided initial mechanistic insight into hemorrhage induction. The work also uncovered several candidate targets for antiviral intervention.

The spleen has been considered a major peripheral lymphoid organ in fish, housing lymphocytes, macrophages, and dendritic cells [29,67]. However, because of differences in taxonomic status, there are variations in splenic histological organization between fish and higher vertebrates, and even among different fish species. A previous study has identified eleven cell clusters in the zebrafish spleen [68]. In the present study, we identified 10 cell populations in the gibel carp spleen, with one population remaining undefined. The marker genes used to identify cell types are mainly from the FishSCT database, a fish scRNA-seq database that contains zebrafish and other fish species [69]. Most classical marker genes, such as *CD3*, *LCK*, and *ZAP70* for T cells, and *CD79* for B cells, are expressed uniquely in the identified cell populations. The newly identified zebrafish eosinophil lineage-specific marker, *ESLEC* [70], showed high-specific expression in gibel carp eosinophils. However, low expression of some marker genes was also observed. For example, the expression of the homologous genes of zebrafish *CDH5* and *KDRL*, which are endothelial cell markers, was low in endothelial cell populations identified in the present study. The cell type was characterized by the unique expression of *PECAM1* and *FLI1*.

For macrophage populations, although 5 cell clusters were identified as macrophages based on UMAP analysis and a heatmap of marker genes, only 3 clusters were observed at day0. Among them, clusters 4 and 9 were the two main macrophage clusters at the time point. The cells of cluster 4 expressed classical macrophage lineage-specific markers, including *CTSS2.2*, *MARCO-A/B*, and *MPEG1*, whereas cluster 9 showed high *CTSS2.2* expression. Cluster 7, a group of virus-infected cells, exhibited marker gene expression patterns similar to those of cluster 4 but at lower levels. Interestingly, *MARCO-A/B* and *MPEG1* expression was low in clusters 9, 10, and 12. The identified feature gene of clusters 10 and 12 is *APOC1*. Although *APOC1* has not been identified as a fish macrophage marker experimentally to date, the gene has been reported to exhibit high expression in specific macrophage subpopulations in humans [57,71], and it promotes macrophage polarization toward the M2 phenotype. The high expression of the gene in clusters 10 and 12 could be associated with the function of the cell group. Our study may also provide a new reference for gene markers in non-model fish. The differences in cell types and marker gene expression between gibel carp and zebrafish could be due to species-specific variation. Nevertheless, we cannot rule out the possibility that discrepancies arise from the scarcity of validated marker genes in non-model fish species.

The present study revealed that macrophages and mural cells are the major splenic cell types infected by CaHV. Macrophages are important components of the innate immune system, with functions including phagocytosis, antigen presentation, tissue repair, and iron homeostasis [72]. Infection of macrophages has been reported in human herpesvirus infections, such as those caused by Epstein-Barr virus (EBV) and VZV [73,74]. These studies also showed that virus-targeted macrophages typically exhibit impaired immune regulation functions—a phenomenon also observed in CaHV-infected macrophages. Thus, targeting innate immune cells, including macrophages, may represent an evolved viral infection strategy. By this strategy, the virus not only directly suppresses the host’s immune response but also exploits the circulatory system to achieve rapid viral dissemination.

Further analysis of macrophages revealed that, under normal physiological conditions, the spleen of gibel carp contains seven distinct macrophage subpopulations corresponding to three cell clusters (4/9/10), in which clusters 9 and 10 constitute Mø2 and Mø5, respectively. Pseudotime and CytoTRACE analysis showed that Mø2 and Mø5 are located at different differentiation states. Enrichment analysis further showed that immune-related genes were highly expressed in Mø2, while marker gene analysis showed that Mø5 exhibited high expression of *ApoE-b* and *ApoC1*, a pattern also observed in Mø6. Combined with cell number variation, we speculated on the infection and differentiation of the Møs. The seven Mø subsets were all infected by CaHV at day 0. Then, the infected cells of Mø1/4/7/9 form Mø3, mainly Mø1 cells, due to virus-mediated changes in gene expression that facilitate virus infection. The antiviral response was strongly activated in Mø2 cells, which maintained the cell subpopulation’s relatively low infection efficiency throughout the infection period. In other words, Mø2 exerted an antiviral function. Mø6 may originate from Mø5 cells, which all possessed anti-inflammatory function. Interestingly, cells with high viral fragment content were not found in the Mø6 subset at day 4. It seems that Mø6 developed resistance to the virus after the early stage of infection, but the mechanisms remain unclear and require further research.

The scRNA-seq data showed that most cells are not infected or are in a low-infection state, even in permissive cell types. For example, more than 60% of macrophages are clustered at a low degree of infection. The previous report has indicated that the host immune responses were initiated or modulated by a small number of infected cells [75]. The transcriptomic changes in infected cells compared to bystander cells would reflect specific responses in the infected cells, which have been observed in viruses including SARS-CoV-2 and African swine fever virus (ASFV) [23,76]. So, we used the method in the present study. Several CaHV-specific responses or -induced genes were identified by the comparison.

A hallmark of CaHV infection is the global suppression of gene expression in infected cells, as evidenced by the downregulation of pathways governing translation and metabolism. Meanwhile, antiviral genes—most notably those in the IFN axis—are also repressed, likely reflecting a viral strategy to facilitate intracellular survival. Two mechanisms may account for this IFN silencing. First, CaHV may encode dedicated antagonists of host antiviral signaling, as documented in herpesviruses infecting mammalian and other species [77–79]. Second, the virus upregulates several endogenous negative regulators of the innate immune response (e.g., *BEND6*) that dampen innate immune responses. These negative immune regulators may represent promising targets for future breeding programs to enhance viral resistance.

However, although antiviral pathways such as the IFN pathway are downregulated in infected cells compared to bystander cells, the overall antiviral response within the permissive cell populations is upregulated, indicating that the viral infection still elicited an antiviral response, which is even stronger in bystander cells. This observation suggests that infected cells release some factors (possibly viral proteins or cytokines) that trigger a robust antiviral response in surrounding cells. A similar phenomenon has been observed in macrophages infected with African swine fever virus (ASFV) [23].

Several pathways, including those related to transport, endocytosis, cell adhesion, and cytoskeletal reorganization, were upregulated, suggesting that these pathways may play important roles in viral infection. We validated two of these pathways. One is the HS pathway, supported by the significant upregulation of *EXTL2*, a key gene involved in HS synthesis [63]. As a receptor mediating virus binding to the cell surface, HS has been utilized by several viruses, including human herpesviruses [80,81]. Our findings highlighted the importance of HS in fish herpesvirus infection and identified the key gene involved. The other pathway is the ESCRT complex-related pathway. The ESCRT machinery comprises several peripheral membrane protein complexes and associated proteins that are crucial for cargo sorting and membrane remodeling and are thus involved in the replication cycles of various human viruses [82]. Our drug-inhibition assay confirmed the important role of the ESCRT complex in CaHV release. However, the detailed interactions between CaHV and the ESCRT complex need further research.

It was observed that both common and distinct responses are induced by CaHV infection in macrophages and mural cells. The distinct responses may be attributed to differences in cell types, as reflected in the DEGs. For the common responses, the pathways related to the negative regulation of immune responses, transport, and endocytosis described above were all significantly enriched in virus-infected cells among the two cell types. In addition, GO terms related to chromatin remodeling and histone acetylation/deacetylation were found in virus-infected cells of both cell types, indicating that changes in chromatin epigenetic modifications or spatial conformations may be required for virus replication. It has been reported that viruses, including herpesviruses, can alter the spatial organization of host genomes, thereby affecting gene expression and virus replication [83].

An interesting phenomenon is the increase in macrophage numbers but a decrease in B cell numbers during infection. Macrophages increased rapidly within 2 days, indicating that the fish rapidly generates macrophages in response to infection stimulation. Then, macrophages are differentiated into distinct subsets that perform different functions. Simultaneously, the virus did not activate a robust cell death pathway in macrophages, thereby maintaining macrophage numbers at the early stage of infection to facilitate virus replication and spread. The decrease in B cell numbers might be a side effect caused by CaHV. The virus secretes factors that damage B cells and suppress the host immune response, thereby facilitating viral infection. It is also consistent with our finding that MHC expression in macrophages is upregulated during viral infection, but viral infection in fish is not inhibited. However, the detailed mechanisms underlying the decrease in B cell numbers need further research.

Hemorrhage is a hallmark clinical sign of many viral infections in aquatic animals. Besides the CaHV examined in this study, similar manifestations are also observed in the hosts infected with grass carp reovirus (GCRV) [84], spring viremia of carp virus (SVCV) [85], and so on. Similar findings have been reported extensively for several viruses that infect humans, such as hemorrhagic fever viruses [86]. In aquatic species, however, the mechanisms underlying virus-induced bleeding remain poorly understood. For GCRV-induced hemorrhage, evidence suggests that complement activation may contribute [84]. Our data reveal that CaHV-induced bleeding is multifactorial, involving both coagulation defects and programmed death of vascular endothelial cells. Endothelial apoptosis has been recognized as the principal cause of hemorrhage in human viral hemorrhagic fevers [86]. Interestingly, vascular endothelial cells are not permissive for CaHV replication, indicating that their cell death is triggered by extrinsic factors released from the permissive cells. Controlling endothelial cell death may therefore represent a promising strategy to mitigate hemorrhage during viral infections.

## Conclusions

**Fig 11:**
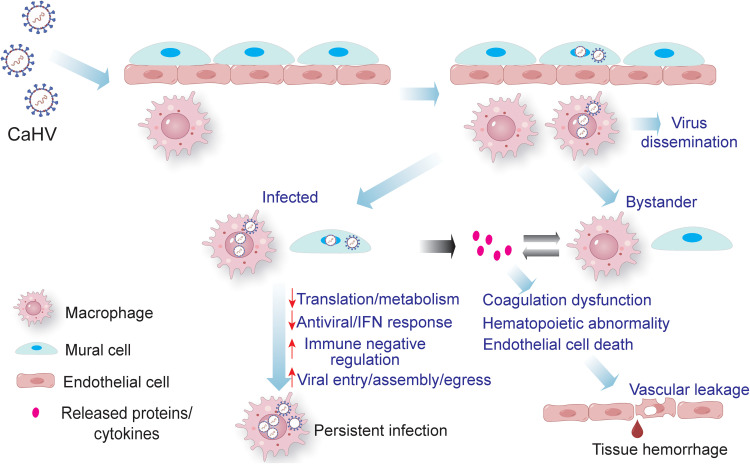
Schematic diagram of the CaHV infection in the spleen of gibel carp. The image was drawn in Adobe Illustrator.

The present study elucidated the splenic transcriptome landscape of gibel carp infected with *Cyvirus cyprinidallo2* at the single-cell level, using CaHV as a model. Further investigations identified the major permissive cell types for CaHV and characterized the virus-host interaction mechanisms (Fig 11). CaHV infects macrophage and mural cells. The infected macrophage in the circulatory system will facilitate viral dissemination throughout the body. Compared with bystander cells, infected cells exhibited downregulated pathways related to translation, metabolism, and antiviral responses, as well as upregulated pathways involved in transport, endocytosis, and so on. These transcriptional alterations may contribute to the establishment of persistent infection. Simultaneously, DEGs of the infected cells were also highly enriched in coagulation and hemotopoietic-related pathways, indicating virus infection induced abnormalities in these biological processes. Additionally, infected cells could release proteins/cytokines originating from the virus or the host, which can stimulate bystander cells and remodel their transcriptomic profiles. These factors from infected/bystander cells trigger programmed cell death of vascular endothelial cells, leading to vascular leakage and subsequent tissue hemorrhage. Several potential antiviral targets were also revealed. Collectively, these findings lay a foundation for deeper elucidation of the pathogenesis of *Cyvirus cyprinidallo2* infection and the discovery of novel antiviral targets.

## Materials and methods

### Ethics statement

All animal experiments were carried out in accordance with the guidelines and regulations of the Animal Research and Ethics Committees of the Institute of Hydrobiology, Chinese Academy of Sciences (protocol code: IHB/LL/2024045). MS-222 was employed for the anesthesia of the fish, and all feasible measures were taken to minimize the discomfort experienced by the fish.

### Animals, cells, virus, and antibodies

Health gibel carp (*C. gibelio*, clone A^+^) with a weight of 19.5 ± 1.5g per fish were obtained from the Liangzi lake station of the Institute of Hydrobiology, Chinese Academy of Sciences. The fish were cultured at 25 °C in tanks with aerated, filtered water for 2 weeks before virus challenge and fed with commercial feed twice a day. Confirmation of individuals with CaHV-free status was performed as described previously [20].

CaHV was preserved in our lab and propagated in gibel carp skin cell line (GiCS) [11,87]. A recently established cell line in our lab, the gibel carp swim bladder cell (GiSB, Chinese patent 202510066699X), was used for gene function analysis due to its relatively high transfection efficiency. The cells were cultured at 25 °C in Leibovitz L-15 (L-15) medium containing 10% fetal bovine serum (FBS).

The rabbit anti-88R polyclonal antibody was prepared by prokaryotic expression of the 250–750 aa of CaHV-88R and immunized rabbits, which was performed by Wuhan Gene Create Biotech Co., Ltd. Mouse anti-Crucian Carp/Goldfish IgM monoclonal antibody (BGD1045, Biogoethe) and rabbit anti-RGS5 polyclonal antibody (11590–1-AP, Proteintech) were purchased from commercial companies. Rabbit anti-IRF3 and anti-Mx polyclonal antibodies were gifts from Prof. Yi-Bing Zhang of the Institute of Hydrobiology, CAS.

### CaHV preparation and animal infection

GiCS cells were infected with CaHV at an MOI of 0.01 and incubated at 25 °C. Cytopathic effect (CPE) reached more than 90% after 9–10 days of incubation. Then, the mixture was collected and centrifuged at 10,000g for 5 min at 4 °C. The supernatants were used as viral stocks for animal infections.

For animal infection, the healthy gibel carps were randomly divided into two tanks with 30 fish per tank. The fish in one tank were intraperitoneally injected with the supernatants obtained above at 200 μL (about 5 × LD_50_, 2 × 10^5^ viral genome copies) per fish. Infection with the dosage will cause major deaths at 5 dpi in our preliminary experiment. The fish of another tank were injected with cell culture medium at the same volume and used as a control. Tissues were collected from the infected fish at 0, 2, and 4 dpi. Nine fish were sampled at each time point. To minimize the impact of individual variability, the spleens of every three fish were pooled into a single sample (n = 3 pools of 3 fish each). Clinical symptoms and survival of the fish were observed daily. Besides the sample collection, the infected fish died at 5 dpi, and the control fish survived.

### Single-cell collection and scRNA-seq

The spleens were washed with PBS, cut into small pieces on ice, and digested in the separation solution (2 mg/mL IV collagenase) in a water bath at 37 °C for 20 min. The mixture was centrifuged at 1,200 rpm for 5min, and the precipitate was then digested with 0.5% trypsin for 10 min at 37 °C. After termination with PBS containing 10% FBS, the cell suspension was filtered with a 40 μm cell strainer and then centrifuged at 300 g for 5 min at 4 °C. The cell pellet was resuspended and subjected to red blood cell removal with red blood cell lysis buffer (Invitrogen, 00–4333). Cell viability of each sample was above 85% by trypan blue exclusion.

Single cell capture of the spleen samples was performed using the 10 × Genomics Chromium Controller system (10 × Genomics, Pleasanton, CA, USA). Briefly, the single cell suspensions were loaded to 10 × Chromium, and cDNA amplification and library construction were performed with 10 × Genomics Chromium Single-Cell 3’ kit (V3) according to the manufacturer’s instructions. The libraries were sequenced at PE150 on the Illumina NovaSeq 6000 sequencing system by Shanghai Personal Biotechnology (Shanghai, China).

### ScRNA-seq data processing

The gibel carp genome reported in a previous study [9] and the CaHV genome [11] were used as reference genomes in the analysis. The raw sequencing reads in FASTQ format were processed using the Cellranger v7.1.0 pipeline (https://www.10xgenomics.com/), including counting, quality control, and mapping with the reference genome.

The gene expression matrices were merged using the Seurat package v5. The following criteria were used in data filtering to collect the usable cells and genes: the gene numbers in a cell should be more than 400 and less than 7000; the total UMI counts in a cell should be less than 50,000; the genes should be expressed in more than 3 cells; mitochondrial gene percentage should be less than 20%. For macrophages, the filtering criterion was set at 25% to prevent the loss of bona fide cells, given their high metabolic activity. The mitochondrial genome (GenBank: KU668576.1) of a wild gibel carp was used in the analysis. The quality control information containing the nCount_RNA, nFeature_RNA, and percent.mito was provided in the S6 Table. The filtered data was normalized using the “LogNormalize” function for data scaling. The top 2,000 highly variable genes (HVGs) were identified with the normalized and scaled data and used in the principal component analysis (PCA) to reduce the dimensionality.

### Cell clustering and differential expression analysis

The top 50 principal components from PCA were used in cell clustering analysis with the UMAP method. DEGs analysis was performed using the Seurat FindMarker function based on the Wilcox likelihood-ratio test with default options. The genes were evaluated as DEGs according to the following criteria: “|avg_log2FC|>1 and pct.1/pct.2>0.25”. GO term and KEGG pathway enrichment analysis of the DEGs were performed with the “clusterProfiler” R package. The significantly enriched functions were determined with an adjust *p*-value < 0.05. The GO terms analyzed in the study are collected in supplementary materials (S7 Table). Gene Set Enrichment Analysis (GSEA) was performed with the GSEA (V1.64.0) computational approach [88].

### Analysis of infected versus bystander cells

To distinguish the cells that are really infected and the ones containing viral UMIs that may be due to ambient contamination, Otsu’s method was used as described previously [23]. The cutoff values were obtained for the infected samples. A cell containing viral UMIs higher than the cutoff was considered “infected”, and the cells with viral UMIs lower than the cutoff were considered “bystander”. The “bystanders” also contained the cells with no viral fragments.

### Gene set score analysis

To evaluate the difference in the central tendencies of a gene set between two independent samples, UCell (V2.3.1) with the Mann-Whitney U statistic was utilized for scoring gene sets.

### Pseudo-time inference

Pseudo-time inference was used to analyze the cell differentiation process and cell fate using the Monocle 2 (version 2.30.1) R package. Data from the identified cell clusters were transferred to the Monocle package to create a new dataset. A cell development trajectory with pseudotime was generated with the functions “reduceDimension” and “orderCells”. DEGs were calculated using the “differentialGeneTest” and “BEAM” functions. We also used the CytoTRACE (V0.3.3) computational framework with default parameter settings to evaluate the initial reference point and the stemness characteristics of cells within the pseudotemporal trajectory analysis.

### Plasmids construction and transfection

To construct plasmids used in the overexpression assay, total RNA was extracted from GiCS cells using the VeZol Reagent (Vazyme, China) according to the manufacturer’s instructions. cDNA was synthesized using HiScript IV 1st Strand cDNA Synthesis Kit (+gDNA wiper) (Vazyme, China). The coding sequences of host genes were amplified using the cDNA as template and ligated into the pcDNA3.1-HA vector using an in-fusion cloning strategy.

For plasmids used in RNA interference, the U6 promoter of PLKO.1 (kindly provided by Prof. Shun Li at the Institute of Hydrobiology, CAS) was first replaced with a U6 promoter from grass carp to obtain the vector PLKO.1-U6-319. Three targets were selected for each gene, and a pair of shRNA oligos was designed and synthesized for each target, according to the protocol described at https://www.addgene.org/protocols/plko/. The oligos were annealed and ligated into PLKO.1-U6-319, which had been predigested with *AgeI* and *EcoRI*. All constructs were verified by DNA sequencing. The primers and constructs were collected in the S8 Table.

The plasmids were transfected into GiSB cells using Lipomaster 3000 Transfection Reagent (Vazyme, China) according to the manufacturer’s instructions. The transfected cells were infected with CaHV 48 h post-transfection. Viral DNA copy numbers were detected after 48 h post-infection. For the RNAi assay, the plasmids were first transfected into GiSB cells, and the knockdown effect of each plasmid was detected, as shown in S8 Fig. Then, the plasmid with the highest inhibitory effect was used in the following transfection and virus infection assay.

### Construction of recombinant CaHV expressing EGFP

The CaHV expressing EGFP was constructed based on homologous recombination as described previously [89]. Briefly, the regions of the CMV promoter and the *EGFP* gene were amplified by PCR using pEGFP-N3 as the template. The two homologous arms (CaHV genome regions 108,129–109,164 bp and 109,165–110,214 bp) were amplified using CaHV genome DNA as a template. The homologous arms (R_L_ and R_R_), CMV promoter, and *EGFP* gene were inserted into the pMD18-T vector by infusion cloning, resulting in the plasmid pMD18T-R_L_-pCMV-EGFP-R_R_. After sequence confirmation by DNA sequencing, the plasmid was transfected into GiCS cells as described above. The cells were infected with CaHV at 12 h post-transfection. Cell medium was collected after the CPE reached about 90% and used to infect fresh GiCS cells. The cells with green fluorescence signals were sorted by flow cytometry and incubated until a total CPE appeared. Then, the cell supernatants were inoculated into fresh GiCS cells, and the cells with green fluorescence were picked by plaque isolation as described previously [89]. A purified recombinant CaHV-EGFP virus was obtained by repeated flow cytometry sorting and plaque isolation. All primers used were collected in the S8 Table.

### Macrophages isolation and culture

Macrophages were isolated from gibel carp spleen using a Percoll-based kit based on a previous report [31]. Briefly, the spleen was removed aseptically, and the cells were collected by passing through a 100 μm mesh in PBS. Then, preliminary macrophages were obtained by centrifugation according to the manufacturer’s instructions, followed by purification through adherence. The isolated cells were cultured under the same conditions as GiCS/GiSB described above. Their morphology and purity were observed by using Wright-Giemsa staining and transmission electron microscopy (TEM). In addition, peripheral blood was collected and used as a control. Expression of marker genes was performed by RT-PCR as described previously [31].

### CaHV-EGFP infection

For the *in vitro* assay, the isolated macrophages above were infected with CaHV-EGFP at 0.1 MOI and incubated at 25 °C. The cells were observed under a fluorescence microscope (Olympus DP80) at different times post-infection. Alternatively, the cells were collected for the detection of virus gene expression and for flow cytometry sorting.

For the *in vivo* assay, healthy gibel carp were intraperitoneally injected with the supernatants of CaHV-EGFP-infected cells as described above. Spleens of the infected or control fish were collected at different time points, and the cells were sorted by flow cytometry based on the green fluorescence signal. The obtained cells were used in the gene expression detection and TEM observation.

### Electron microscopy

Virus-infected or control fish spleen/cells were collected and fixed with 2.5% glutaraldehyde. Ultrathin sections were prepared using the methods described previously [87]. Examination was performed with a Hitachi HT-7700 transmission electron microscope (TEM) at 80 KV. For negative staining, the virions in supernatants of the treated or untreated cells were concentrated by ultracentrifugation and resuspended in the same volume of PBS. Negative staining was performed as described previously [90], and observed as above.

### Western blotting

Western blotting was performed as described previously [89]. Briefly, the samples were analyzed by a 4–20% SDS-PAGE gel and transferred to a PVDF membrane (Millipore). The polyclonal antibodies were used as the primary antibody. The horseradish peroxidase (HRP)-conjugated goat anti-rabbit IgG(H + L) (Abclonal) was used as the secondary antibody. Antibody binding was examined by chemiluminescence (Millipore).

### Virus binding and infection assays

For virus binding, GiSB cells were pre-seeded into 24-well plates and incubated with CaHV at an MOI of 1 for 1h at 4 °C, with or without different treatments. Then, the cells were washed three times with L-15 medium and harvested for qPCR analysis. For virus infection, the pre-seeded GiSB cells were infected with CaHV at an MOI of 0.1 at 25 °C, and CPE was observed under a microscope at different time points with the corresponding treatment.

### Chemical treatment assay

For the heparin or heparan sulfate (HS) incubation assay, different concentrations (0, 0.1, 1, 10, and 100 mg/mL) of heparin or HS were preincubated with CaHV suspension at 4 °C for 1h, and then the mixtures were incubated with GiSB cells in the virus binding and infection assays as described above.

For the heparinase treatment assay, GiSB cells were treated with heparinase I (*Flavobacterium heparinum*) at different concentrations (0, 1, 2, and 5 U/ml) at 25 °C for 1h. Then, the cells were washed and used in the virus binding and infection assays.

For DBeQ and tenatoprazole treatment, the cytotoxicity of the two drugs was assessed using a CCK-8 assay before the experiment. GiSB cells were infected with CaHV at 0.1 MOI for 96 h. Then, the cell culture medium was replaced with that containing DBeQ (6 μM) or tenatoprazole (100 μM). The DBeQ-containing medium was removed after 1h of treatment, and fresh medium was added. The cell medium supernatants with the two drug treatments were both collected at 48 h after the treatment to detect the virions released into the supernatants.

### Detection of viral copy numbers

Samples of the above were collected, and DNA was extracted using the phenol-chloroform method. Viral DNA copy numbers were detected by qPCR with a pair of primers targeting the CaHV major capsid protein gene *88R*. qPCR was conducted in a Quantagene q225MX Real-Time PCR Detection System (Kubo Tech, China). Each qPCR mixture possessed 5 µL of SYBR Premix (2×), 0.5 µL of forward and reverse primers (for each primer), 1 µL of DNA, and 3 µL of ultrapure water. The plasmid pcDNA3.1-88R, constructed in our previous study, was used to generate the standard curve. The qPCR conditions were as follows: 95 °C for 10 min; 40 cycles of 95 °C for 15 s, 60 °C for 1 min; and a melt curve analysis at 95 °C for 15 s, 60 °C for 1 min, and 95 °C for 15 s.

### H&E and TUNEL staining

The spleen and gill were collected at different time points, fixed in 4% paraformaldehyde, dehydrated, and paraffin-embedded. The embedded tissues were sectioned, deparaffinized, and stained with hematoxylin and eosin successively. After dehydration and mounting, the sections were observed under a microscope.

TUNEL staining was performed using a TUNEL kit (Roche, 11684817910). Briefly, the deparaffinated sections obtained above were first treated with citrate antigen retrieval buffer (pH 6.0). Then, they were blocked with 3% goat serum for 30 min and incubated overnight with reagent 1 (TdT) and reagent 2 (dUTP). The sections were washed, stained with DAPI, and mounted with antifade mounting reagent. Observations were performed under a fluorescence microscope (Nikon Eclipse).

### Immunohistochemistry assay

Antigen retrieval of the deparaffinated sections was performed as described above. The sections were then blocked with 3% goat serum and incubated with the primary antibodies overnight at 4 °C. After washing with PBS, the sections were incubated with the corresponding secondary antibody for 1 h at room temperature, and then washed and stained with DAPI. For the two primary antibodies came from the same species (rabbit), the tyramide signal amplification method was used as described [91]. Observations of the sections were performed as above.

### Statistical analysis

The statistical analysis was performed using GraphPad Prism 8. The significance of the difference between groups was analyzed with the Student’s t-test. Data were presented as the mean ± standard deviations from three independent experiments.

## Supporting information

S1 Fig Violin plot showing the proportion of cells containing viral fragments in each cell type at different time points. (TIF)

S2 Fig GO terms enrichment analysis of DEGs of macrophages in the day4 vs day0 group (A) and the up-regulated DEGs (B) or down-regulated DEGs (C) in infected vs bystander group at 4 dpi. (TIF)

S3 Fig Macrophage subsets proportions and MHC genes expressions. (A) Proportions of the nine macrophage subsets at different time points. (B) Expression level of MHC-related genes in different macrophage subsets based on scRNA-seq data, which were displayed as violin plot of the gene expressions in each cell subpopulations at different time point.
(TIF)

S4 Fig CytoTRACE analysis of the nine macrophage subsets and their relationship with the originally identified cell clusters. (A) CytoTRACE analysis of the nine macrophage subsets. (B) Correspondence between the nine macrophage subpopulations (Mø1- Mø9) and the originally identified cell clusters (0–15) in UMAP analysis at different time points. The cell clusters were listed in brackets and that with larger quantities was placed first.
(TIF)

S5 Fig GO terms enrichment analysis of the upregulated DEGs (A) and downregulated DEGs (B) in the day2 vs day0 group of mural cells. (TIF)

S6 Fig GO terms enrichment analysis of the upregulated DEGs (A) and downregulated DEGs (B) in the day4 vs day0 group of mural cells. (TIF)

S7 Fig Subpopulation of mural cells. (A) Heatmap of the top 30 marker genes from the mural cell subpopulations. (B) Cell populations of the mural cell subsets identified with marker genes at different time points.
(TIF)

S8 Fig Detection of the inhibition effects of different shRNAs to the targeted gene. The shRNA possessing the highest inhibition effect was used in the virus infection and detection assay.
(TIF)

S1 Table Basic information of the scRNA-seq data. (XLS)

S2 Table Cell numbers of Macrophage subsets at different time point. (XLS)

S3 Table The proportion of viral UMIs in Macrophage subsets at day2. (XLS)

S4 Table The proportion of viral UMIs in Macrophage subsets at day4. (XLS)

S5 Table Average expression level of genes in macrophages subsets in cell trajectory analysis. (XLS)

S6 Table Basic information of the quality control of the scRNA-seq data. (XLS)

S7 Table GO terms analyzed in the study. (XLS)

S8 Table Primers and constructed plasmids in the study. (XLS)
