# Supplementary figures and images for: Single-cell transcriptome profiling reveals herpesviral manipulation of host processes in spleen of gibel carp infected with a Cyvirus cyprinidallo2
Source: PLoS Pathog. 2026 Apr 2;22(4):e1014114. doi: 10.1371/journal.ppat.1014114 (PMC13061325; doi:10.1371/journal.ppat.1014114)

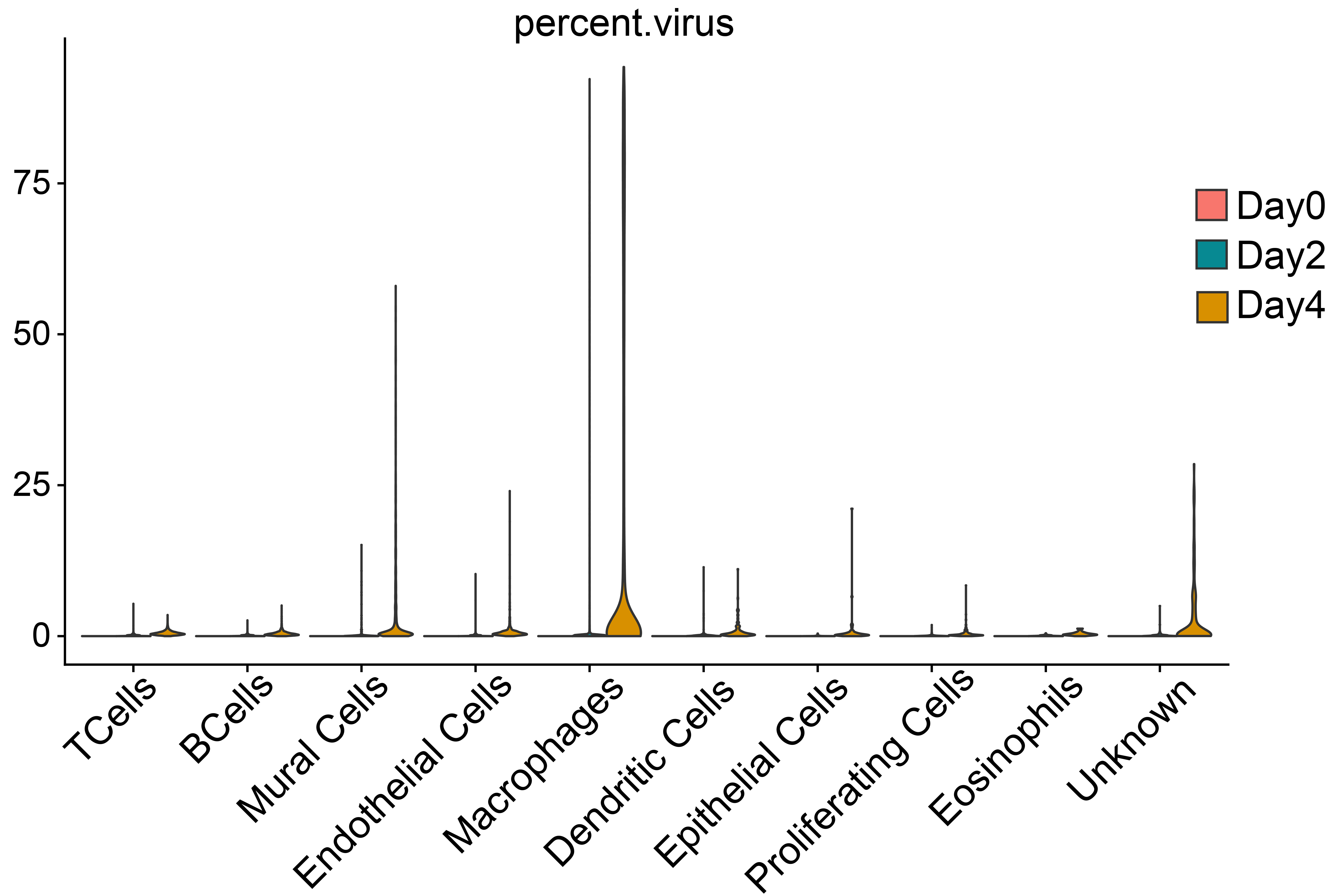

Supplement: S1 Fig — (TIF) [file ppat.1014114.s001.tif]

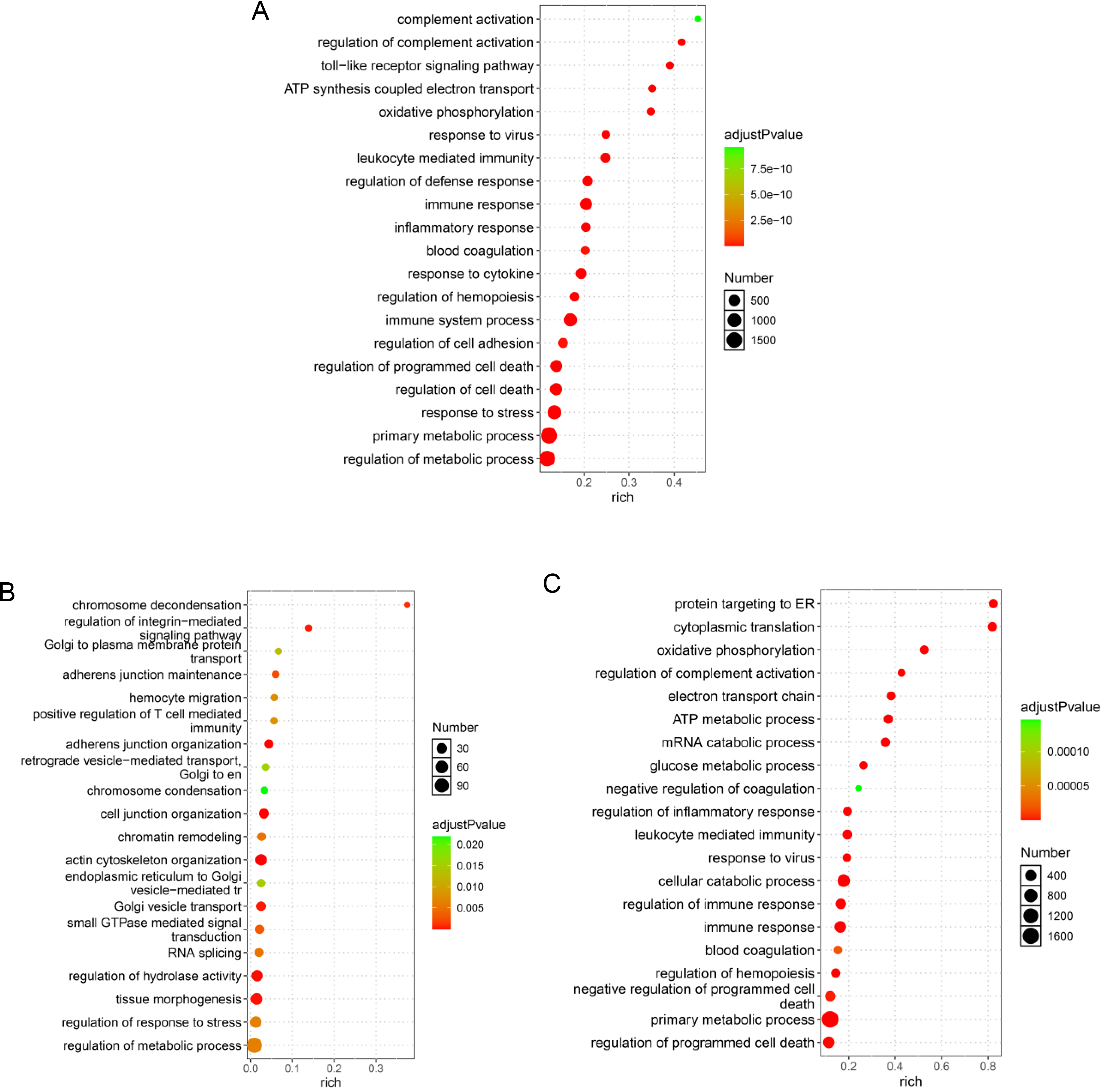

Supplement: S2 Fig — (TIF) [file ppat.1014114.s002.tif]

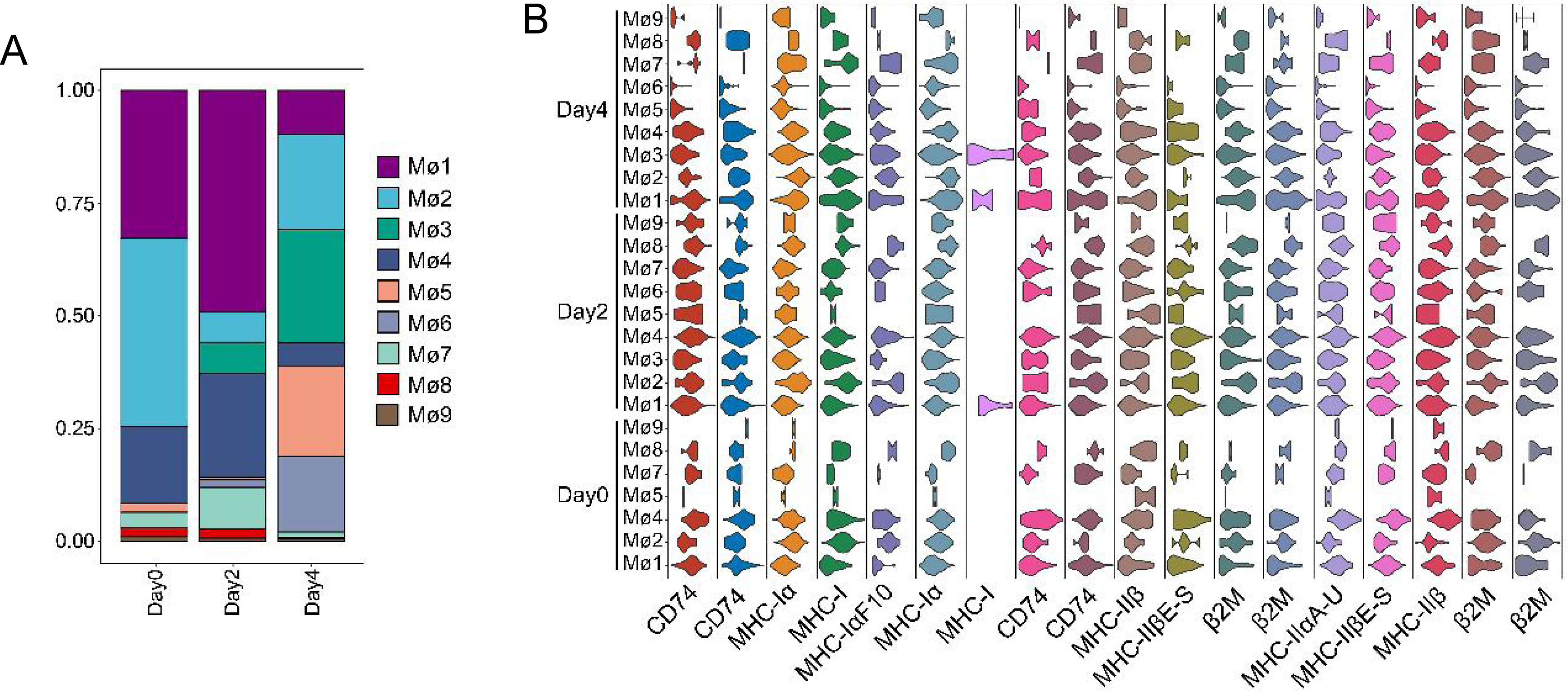

Supplement: S3 Fig — (A) Proportions of the nine macrophage subsets at different time points. (B) Expression level of MHC-related genes in different macrophage subsets based on scRNA-seq data, which were displayed as violin plot of the gene expressions in each cell subpopulations at different time point. (TIF) [file ppat.1014114.s003.tif]

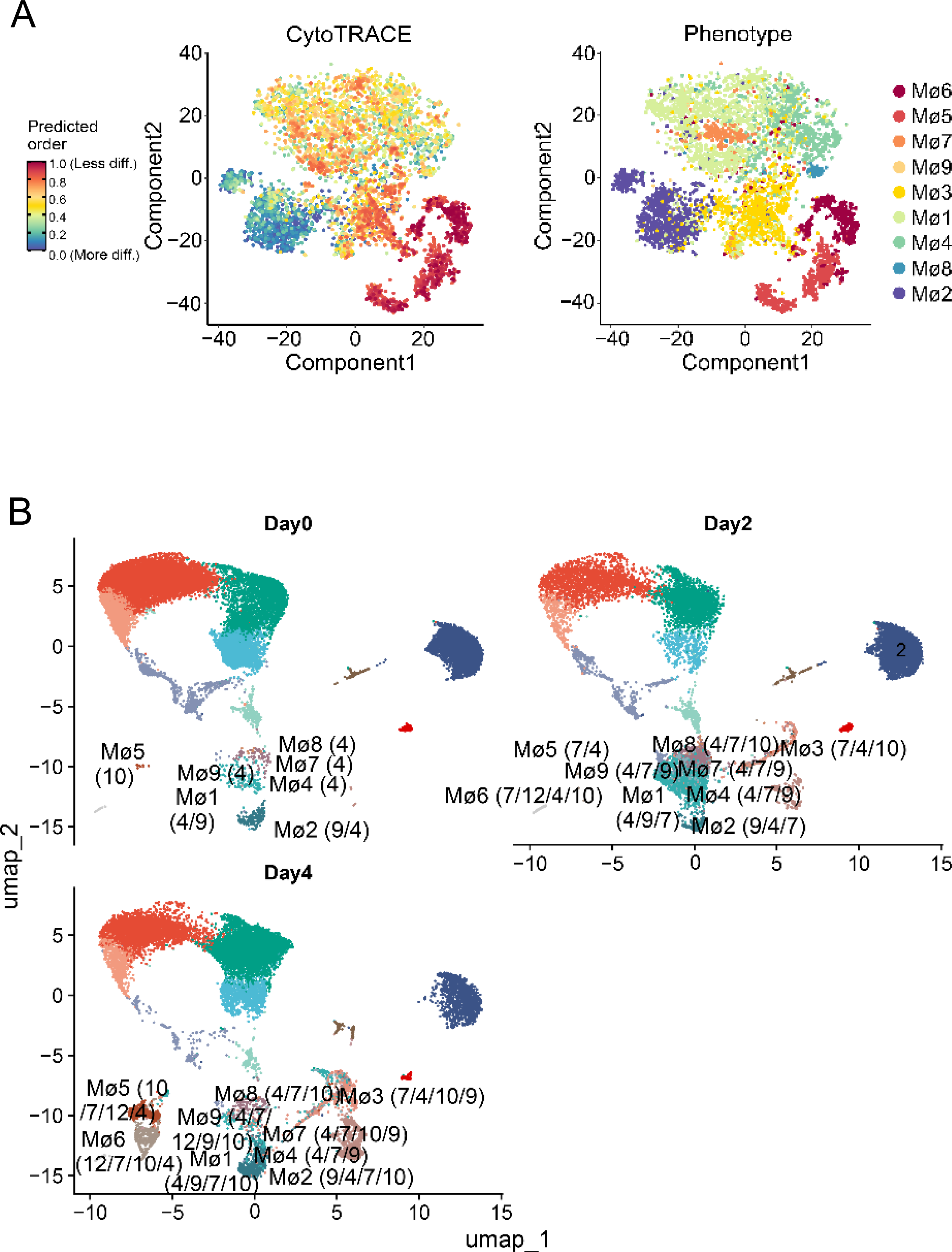

Supplement: S4 Fig — (A) CytoTRACE analysis of the nine macrophage subsets. (B) Correspondence between the nine macrophage subpopulations (Mø1- Mø9) and the originally identified cell clusters (0–15) in UMAP analysis at different time points. The cell clusters were listed in brackets and that with larger quantities was placed first. (TIF) [file ppat.1014114.s004.tif]

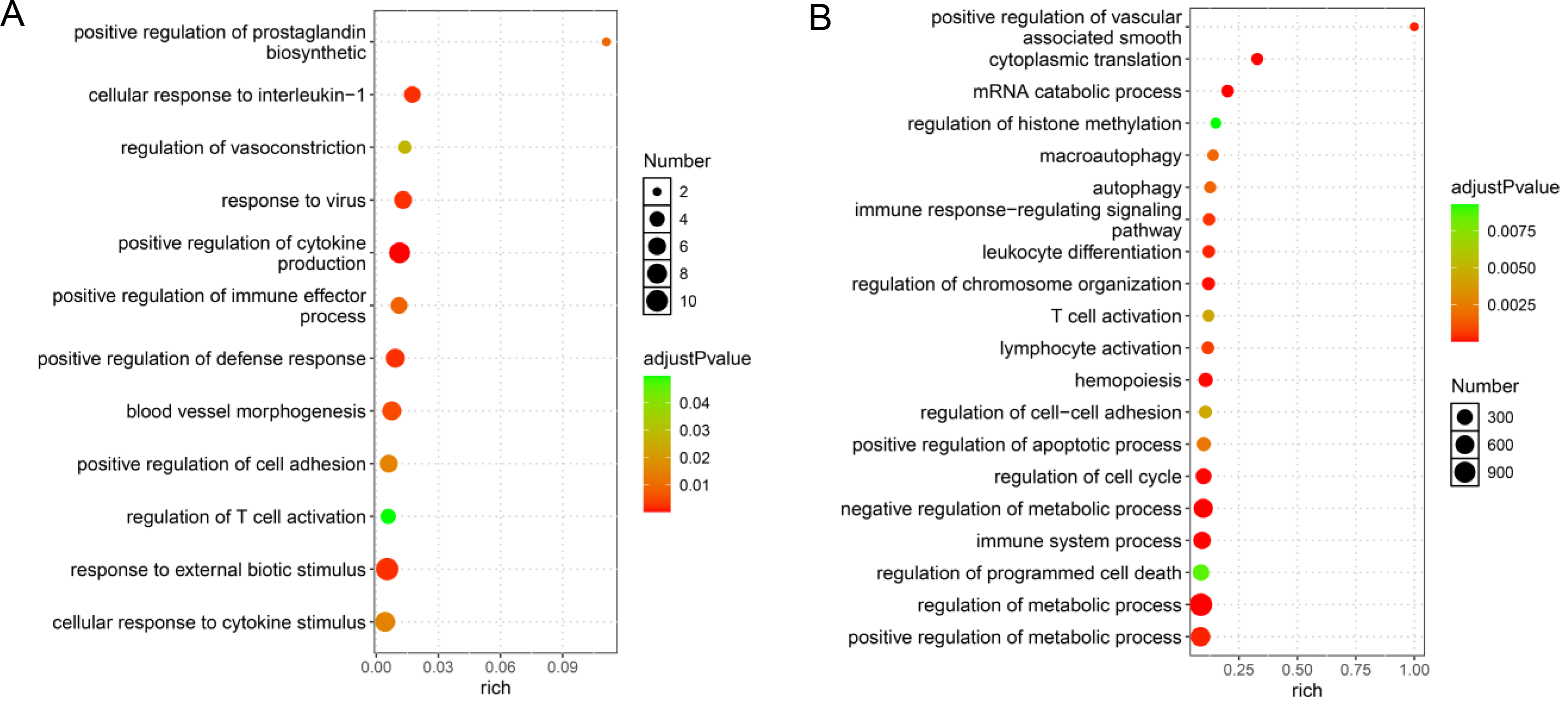

Supplement: S5 Fig — (TIF) [file ppat.1014114.s005.tif]

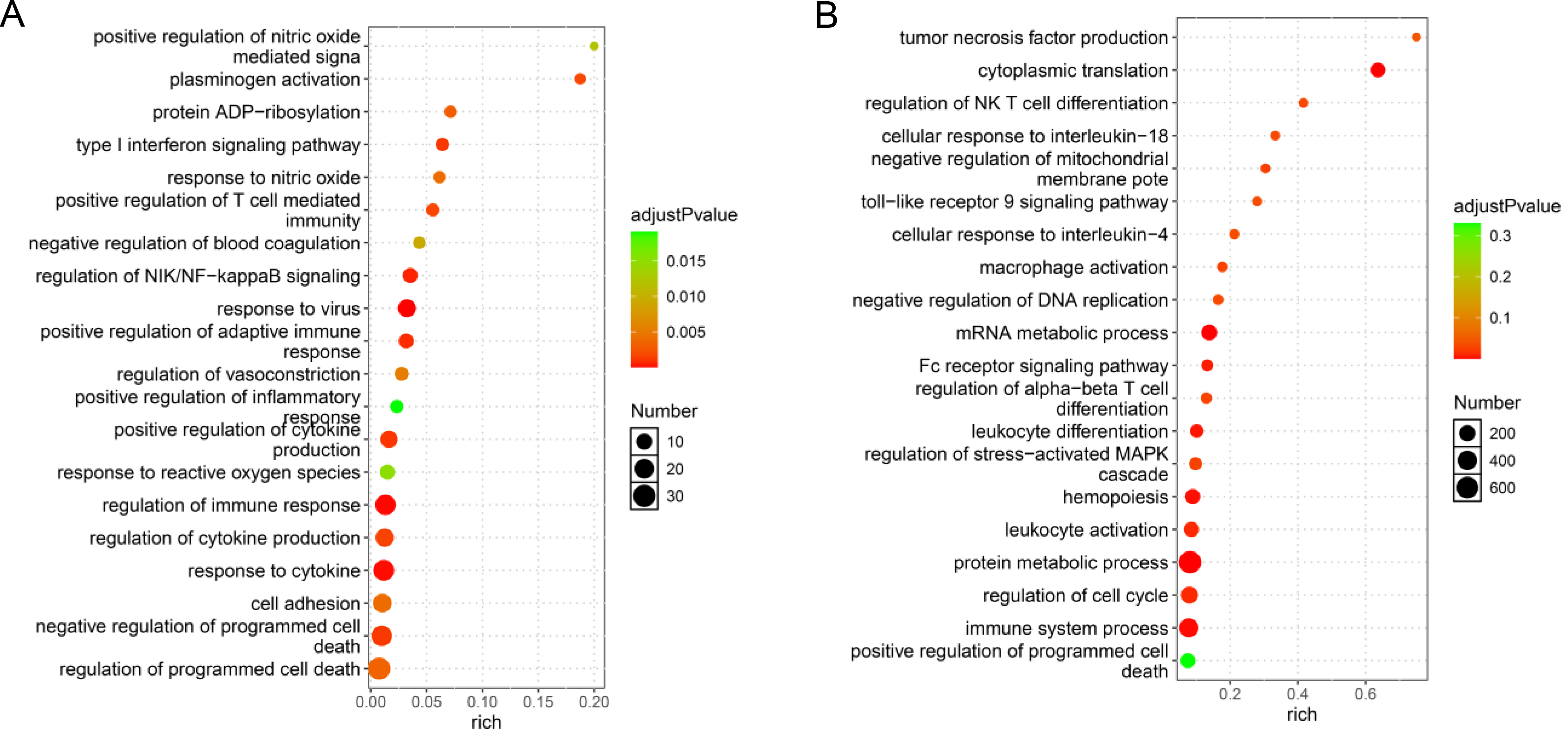

Supplement: S6 Fig — (TIF) [file ppat.1014114.s006.tif]

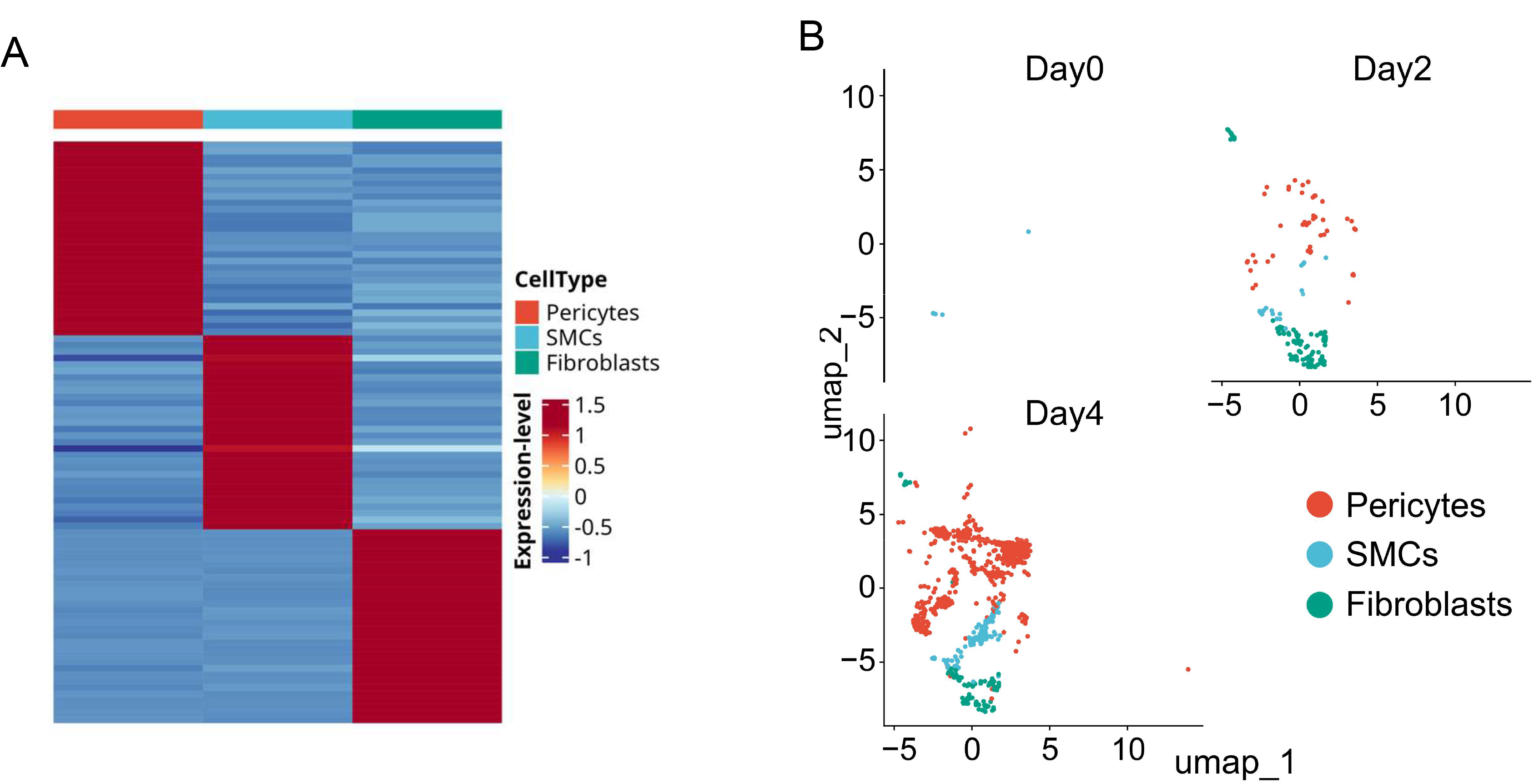

Supplement: S7 Fig — (A) Heatmap of the top 30 marker genes from the mural cell subpopulations. (B) Cell populations of the mural cell subsets identified with marker genes at different time points. (TIF) [file ppat.1014114.s007.tif]

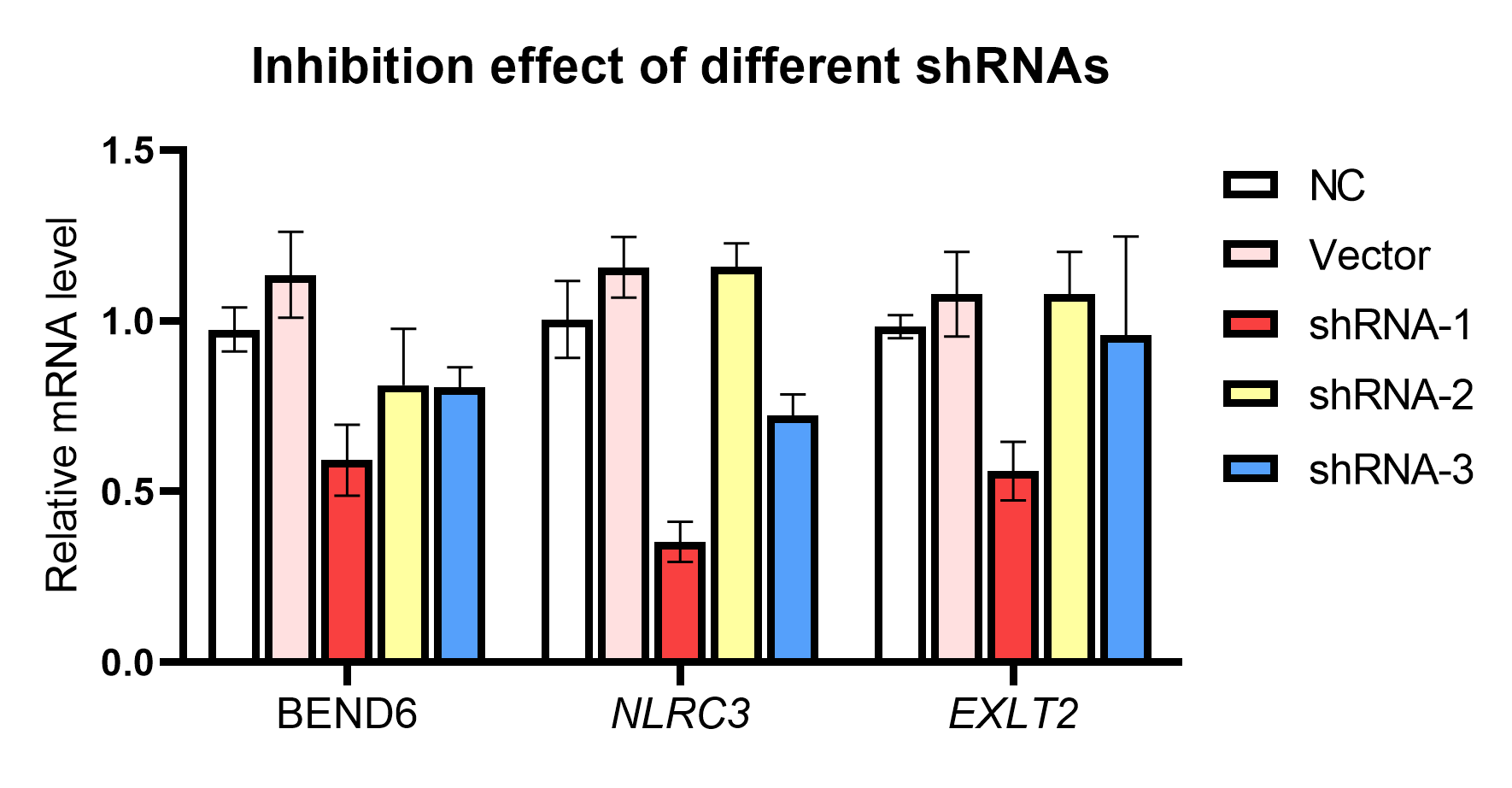

Supplement: S8 Fig — The shRNA possessing the highest inhibition effect was used in the virus infection and detection assay. (TIF) [file ppat.1014114.s008.tif]
